# The Viromes of Six Ecosystem Service Provider Parasitoid Wasps

**DOI:** 10.3390/v15122448

**Published:** 2023-12-16

**Authors:** Gabriela B. Caldas-Garcia, Vinícius Castro Santos, Paula Luize Camargos Fonseca, João Paulo Pereira de Almeida, Marco Antônio Costa, Eric Roberto Guimarães Rocha Aguiar

**Affiliations:** 1 Virus Bioinformatics Laboratory, Centro de Biotecnologia e Genética, Universidade Estadual de Santa Cruz, Ilhéus 45662-900, Brazil; gabrielabcg@gmail.com (G.B.C.-G.); camargos.paulaluize@gmail.com (P.L.C.F.); 2 Department of Biochemistry and Immunology, Instituto de Ciências Biológicas, Universidade Federal de Minas Gerais, Belo Horizonte 30270-901, Brazil; vini8cs@gmail.com (V.C.S.); joaopaulobio@ufmg.br (J.P.P.d.A.); 3 Department of Genetics, Instituto de Ciências Biológicas, Universidade Federal de Minas Gerais, Belo Horizonte 30270-901, Brazil; 4 Departament of Biological Sciences, Universidade Estadual de Santa Cruz, Ilhéus 45662-900, Brazil; costama@uesc.br

**Keywords:** virus, biological control, insect, Hymenoptera, Aculeata, natural enemy, RNA-seq, *Braconidae*, Crabronidae

## Abstract

Parasitoid wasps are fundamental insects for the biological control of agricultural pests. Despite the importance of wasps as natural enemies for more sustainable and healthy agriculture, the factors that could impact their species richness, abundance, and fitness, such as viral diseases, remain almost unexplored. Parasitoid wasps have been studied with regard to the endogenization of viral elements and the transmission of endogenous viral proteins that facilitate parasitism. However, circulating viruses are poorly characterized. Here, RNA viromes of six parasitoid wasp species are studied using public libraries of next-generation sequencing through an integrative bioinformatics pipeline. Our analyses led to the identification of 18 viruses classified into 10 families (*Iflaviridae, Endornaviridae, Mitoviridae, Partitiviridae, Virgaviridae, Rhabdoviridae, Chuviridae, Orthomyxoviridae, Xinmoviridae,* and *Narnaviridae*) and into the *Bunyavirales* order. Of these, 16 elements were described for the first time. We also found a known virus previously identified on a wasp prey which suggests viral transmission between the insects. Altogether, our results highlight the importance of virus surveillance in wasps as its service disruption can affect ecology, agriculture and pest management, impacting the economy and threatening human food security.

## 1. Introduction

The study of insect viruses holds significant value for numerous reasons. Firstly, it enables the prediction of the risk of pathogenic virus spillover events that could harm economically important beneficial insects and endemic species [1]. Additionally, it aids in solving issues related to viral taxonomy and evolution [2,3] as well as understanding the evolutionary relationships between viruses and their hosts [4]. Furthermore, it may facilitate the identification of causal agents of disease outbreaks in animals and plants [5]. It should be noted that research on insect viruses has primarily focused on medical research, including hematophagous insect species that directly transmit vector-borne diseases [6]. Nevertheless, insects are the most diverse group of the animal kingdom, with approximately 5.5 million species on Earth [7]. Despite over one million insect species being scientifically identified, there are significant knowledge and research gaps concerning their viromes.

Parasitoid wasps are essential insects for the biological control of agricultural pests [8]. They lay eggs inside the body of their arthropod hosts, where the immature offspring will develop and ultimately kill their parasitized prey [9]. In addition, they can pollinate, disperse seeds, and help in the decomposition and recycling of nutrients of vertebrate dead bodies (reviewed in [10]). Nonetheless, global insect decline has been intensively evidenced in the last few decades [11,12], and little attention has been paid to this group of insects. In 2018, a declining trend for 11 out of 48 (23%) cuckoo wasp species in Finland was described, probably due to habitat loss [13]. However, important ecosystem service providers of Hymenoptera, like predatory and parasitoid wasps, are still understudied [14]. 

**Figure 1:**
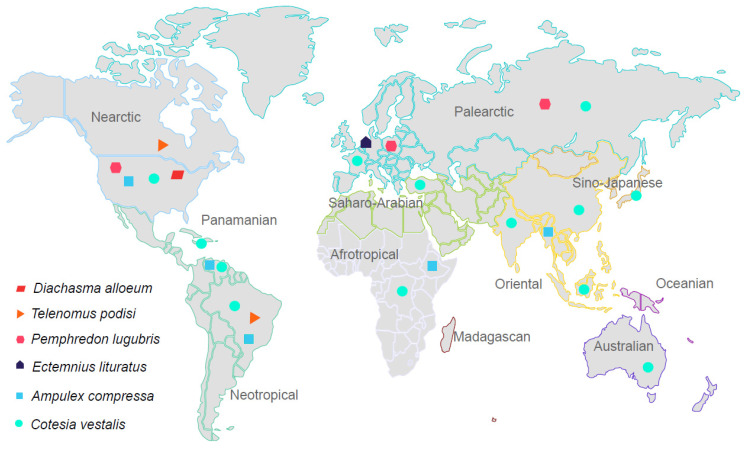
Map of the geographical distribution of the six selected parasitoid species. Each symbol represents a wasp species and refers to the large biogeographical regions instead of single countries. The zoogeographic realms and regions were divided and are represented according to Holt et al., 2013.

To begin unraveling the viral diversity and dynamics in parasitoid wasps, six species widely distributed across the planet were selected for this study (Figure 1). First, *Ampulex compressa* (*Ampulicidae*), native to Ethiopian and Oriental regions [15], is a specialist parasitoid of the American cockroach *Periplaneta americana* [16], a cosmopolitan invasive household pest. The second is *Cotesia vestalis* (*Braconidae*), a specialist endoparasitoid of the diamondback moth (*Plutella xylostella*), which is a serious pest of brassica vegetables of global concern [9]. This host of *C. vestalis* larvae causes critical economic losses worldwide [17]. Third, *Diachasma alloeum* (*Braconidae*) is a parasitoid of the apple maggot fly (*Rhagoletis pomonella*), which is an economically important agricultural pest of apple crops in North America [18]. Fourth, *Ectemnius lituratus* (*Crabronidae*) is a parasitoid digger wasp from Europe that nests in burrows in a variety of dead wood. Flies of medium size are collected as prey to nourish nest cells [19]. Fifth, *Pemphredon lugubris* (*Crabronidae*) is a parasitoid digger wasp widely distributed throughout the northern hemisphere. It nests in dead and decaying wood [20]. Its prey is primarily aphids, which are very damaging pests of a wide range of crops of economic importance, including cereal crops [21,22]. Finally, *Telenomus podisi* (*Platygastridae*), an egg-parasitoid wasp found in Brazil and in the United States [23], has been shown to be important in controlling its preferential host *Euchistus heros,* an abundant pest of soybean crops [24,25]. 

Previous research focused on parasitoid wasps’ virome revealed the presence of members of *Reoviridae*, *Iflaviridae* [26], *Dicistroviridae* [27,28], *Lispiviridae*, *Rhabdoviridae* [29], and *Nyamiviridae* [30]. However, exogenous RNA viruses of these insects are still poorly investigated. On the other hand, parasitoid wasps have been substantially studied with regard to the endogenization of viral elements and the transmission of endogenous viral proteins that facilitate parasitism; thus, the integration of viral sequences into their genome is a well-explored process [4,31,32,33,34,35,36]. Endogenous viral elements have functional roles in the parasitism success of these wasps, as well as being inheritable through wasp generations, an example of convergent evolution between DNA viruses and their hosts [37]. 

This study aims to investigate the viral diversity of six parasitoid wasps through the analysis of fourteen publicly available RNA deep sequencing libraries. The data provided in our study highlight the importance of virus surveillance in wasps as its service disruption can affect ecology, agriculture, and pest management, impacting the economy and threatening human food security. 

## 2. Materials and Methods

- Recovery and processing of RNA-seq libraries

Paired-end public libraries of long RNAs from six species of parasitoid wasps (*Ampulex compressa*, *Cotesia vestalis*, *Diachasma alloeum*, *Ectemnius lituratus*, *Pemphredon lugubris,* and *Telenomus podisi*) were retrieved from the NCBI Sequence Read Archive (SRA) repository (https://www.ncbi.nlm.nih.gov/sra), accessed on 4 October 2022. The accession numbers of the 14 selected libraries grouped by species are *A. compressa* (SRR14607675), *C. vestalis* (SRR6706566, SRR13704978, SRR13704979, SRR13704980, SRR13704991, SRR13704992, and SRR13704971), *D. alloeum* (SRR2041626 and SRR2040481), *E. lituratus* (ERR6054901), *P. lugubris* (ERR8571638), and *T. podisi* (SRR1274857 and SRR1274858). In addition, the geographical origin of the samples used for RNA library construction is represented in Supplementary Figure S1 and Supplementary Table S1. All libraries were processed using tools implemented on the Galaxy Australia web-based platform [38]. The raw reads’ quality was assessed through FastQC version 0.73 [39]. Then, for quality filtering, Illumina adapters and bases with poor quality scores (<20) were removed by sliding window trimming operation in Trimmomatic v. 0.36.6 [40]. The remaining reads were mapped against each wasp genome (except *T. podisi* which does not have an available reference genome) using Bowtie2 v. 2.4.5 [41]. The unaligned sequences were used as input to assemble the putative viral genomes with SPAdes v. 3.15.4 [42] (Supplementary Table S1). Furthermore, the assembled transcripts were queried against the complete viral RefSeq (protein) database retrieved from the NCBI (https://ftp.ncbi.nlm.nih.gov/refseq/release/viral/), accessed on 6 October 2022, and uploaded to Galaxy using DIAMOND BLASTx v. 2.0.15 [43], setting E-values ≤ 10^−5^ and standard parameters. For the metagenomics analyses, TaxonKit [44] was applied. An overview of the sequence similarity results is available in Supplementary Table S2.

- Manual curation and improvement of putative viral genomes

We discarded all hits showing similarity to DNA viruses, retroviruses, and transposons since they mostly represent false positives in virome studies. The remaining sequences were double-checked regarding their viral origin using the online NCBI BLAST (https://blast.ncbi.nlm.nih.gov/Blast.cgi) (accessed on 7 October 2022), specifically BLASTn (nucleotide collection database) and BLASTx (non-redundant protein sequences database). The sequences showing similarity to RNA viruses were further analyzed. For each putative viral sequence, genomic RNAs were manually inspected using Expasy translate (https://web.expasy.org/translate/), and ORFfinder (https://www.ncbi.nlm.nih.gov/orffinder/) (accessed on 10 October 2022). The ORFs larger than 100 nt were chosen for the analyses of conserved domains using profile hidden Markov models with HMMER (https://www.ebi.ac.uk/Tools/hmmer/) (accessed on 10 October 2022) [45] and NCBI Conserved Domains search tools https://www.ncbi.nlm.nih.gov/Structure/cdd/wrpsb.cgi (accessed on 10 October 2022) [46]. Putative viral sequences that did not match the length or ORF structure in comparison to their closest relative in the NCBI nucleic acid databases were further investigated, and libraries from which the virus genome originated were submitted to a new round of assembly using an integrative strategy composed of multiple assemblers (SPAdes [42], Trinity [47], metaSPAdes [48], rnaviralSPAdes, metaviralSPAdes v. 3.15.5 [49], Oases v. 0.1.2 [50], and MEGAHIT v. 1.2.9 [51]) followed by transcript consolidation with Cap3 [52] as described by Espinal et al., 2023 [53].

- Phylogenetic analyses

For the phylogenetic analyses, we first selected complete genomes (length ≥ 90% of related viruses’ genome size); second, fragments of viral genomes (length ≥ 500 nt, which were identified as RNA-dependent RNA polymerase (RdRp) by BLAST and presented conserved domains); third, other RdRp fragments (length ≥ 1000 nt, with the longest ORF ≥ 70% of the full-size sequence, with conserved domains). For the construction of phylogenetic trees, we obtained related virus sequences using BLASTn or BLASTx, according to the highest percentages of similarity and query coverage. The trees of putative new viruses were built with protein sequences that we retrieved from online BLASTx in association with sequences of viruses recognized by the International Committee on Taxonomy of Viruses (ICTV) when possible. The species were selected based on closely related genera or members of the same taxonomic family. Members of other families or distant genera, classified by the ICTV, were chosen as outgroups. More details on the phylogenetic trees’ construction are available in Supplementary Table S3. The selected ORFs (aa) or complete genome sequences (nt) were aligned by MAFFT (https://www.ebi.ac.uk/Tools/msa/mafft/) (accessed on 11 October 2022). Minimal manual adjustments and end trimming were carried out using AliView v. 1.28 [54]. The statistical selection of best-fit models of nucleotide and protein substitution was determined based on the Akaike information criterion (AIC) using ModelTest-NG on XSEDE v. 0.1.7 [55]. After, maximum likelihood trees were inferred by RAxML-HPC BlackBox v. 8.2.12 [56], with 1000 bootstrap replicates. Both tools were implemented on CIPRES Science Gateway [57]. Finally, the generated phylogenetic trees were visualized and edited with FigTree v. 1.4.4 [58] and iTol v.6.7.2 [59].

- Quantification of viral sequences

The transcriptome assembled for each wasp species was evaluated with TransDecoder (https://github.com/TransDecoder/TransDecoder) (accessed on 15 March 2023) to identify the most likely coding sequences (CDSs). The resulting host transcripts were then added to the assembled viral transcripts to estimate the viral abundance in comparison to host mRNAs using the software Salmon v.1.9.0 [60]. For comparison with viral quantification, endogenous and standard genes were chosen; the hosts’ mitochondrial cytochrome b (*cytb*) and nuclear calmodulin were identified via sequence similarity searches (by BlastN) using *Vespa velutina* orthologous genes (MW401001.1 and XM_047509146.1, respectively) as references.

## 3. Results

### 3.1. Metagenomics Analyses

**Figure 2:**
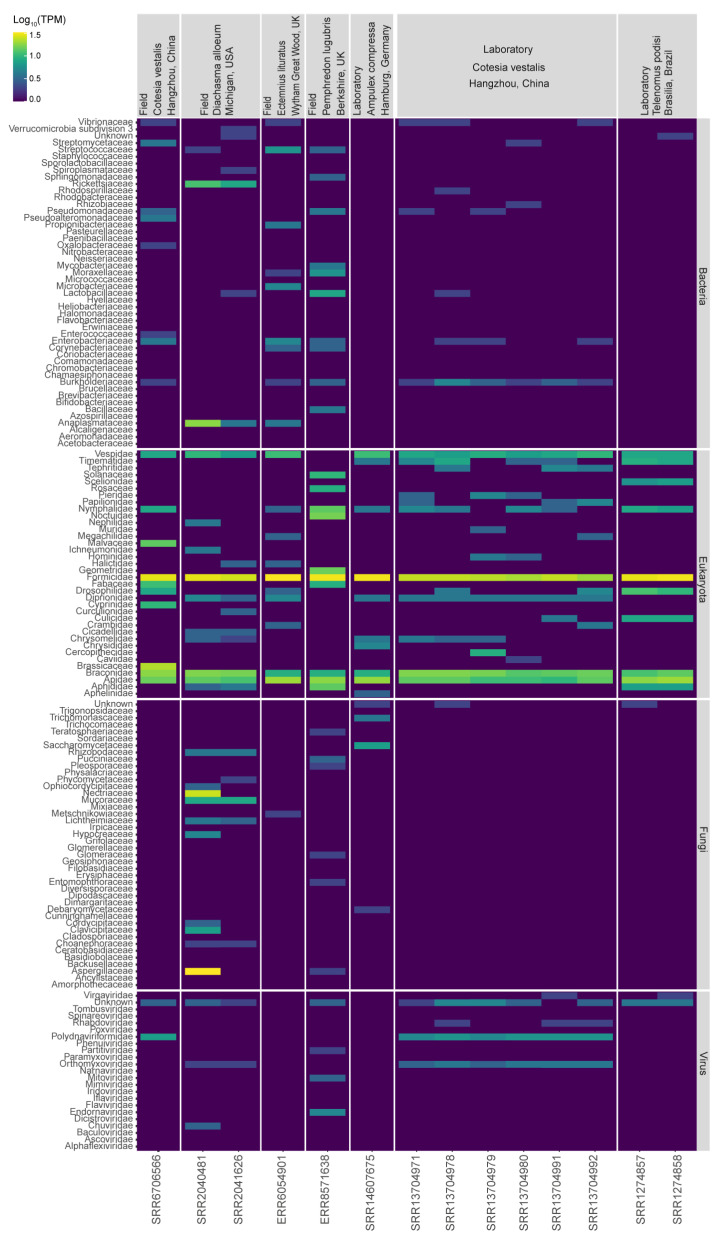
Metatranscriptomics analysis for the six parasitoid wasps investigated in our study. Taxonomic classifications for bacteria, Eukaryota, fungi, and viruses are given by the library and wasp species. The geographical origins and collection sites are indicated in the top rows.

The initial metagenomics analyses revealed the presence of genetic material derived from several microorganisms classified into different kingdoms. As expected, we detected several transcripts matching Eukaryotic species, with higher abundance for transcripts matching insect sequences likely due to the lack of well annotated reference genomes for the species analyzed (Figure 2). We also observed transcripts derived from Bacteria in all libraries analyzed (*A. compressa*: 4%; *C. vestalis*: 7.1–10.1%; *D. alloeum*: 11.4%; *E. lituratus*: 21.1%; *P. lugubris*: 14.2%; and *T. podisi*: 2.7%). Overall, the most frequent families of bacteria were *Streptomycetaceae, Pseudomonadaceae, Lactobacillaceae, Enterobacteriaceae, Burkholderiaceae, Rickettsiaceae,* and *Anaplasmataceae* (Figure 2). In addition, we detected Fungal transcripts (*A. compressa*: 8.9%; *C. vestalis*: 0.5–2.4%; *D. alloeum*: 35.7%; *E. lituratus*: 4%; *P. lugubris*: 5.8%; and *T. podisi*: 1.8%). Some of the fungal families identified were: *Saccharomycetaceae, Nectriaceae, Mucoraceae, Hypocreaceae, Glomerellaceae, Clavicipitaceae,* and *Aspergillaceae* (Figure 2). Finally, transcripts showing similarity with viruses were distributed among *Endornaviridae, Mitoviridae, Partitiviridae, Narnaviridae, Iflaviridae, Rhabdoviridae, Orthomyxoviridae, Chuviridae, Phenuiviridae*, and *Virgaviridae* (*A. compressa*: 1%; *C. vestalis*: 3.5–9.4%; *D. alloeum*: 2.4%; *E. lituratus*: 0.8%; *P. lugubris*: 3.1%; and *T. podisi*: 2.9%). Detailed results grouped by species or for each library are shown in Supplementary Figures S2 and S3. The transcripts showing similarity to viruses were further analyzed.

### 3.2. Characterization of the Wasps’ Virome

- ***Ampulex compressa***

**Figure 3:**
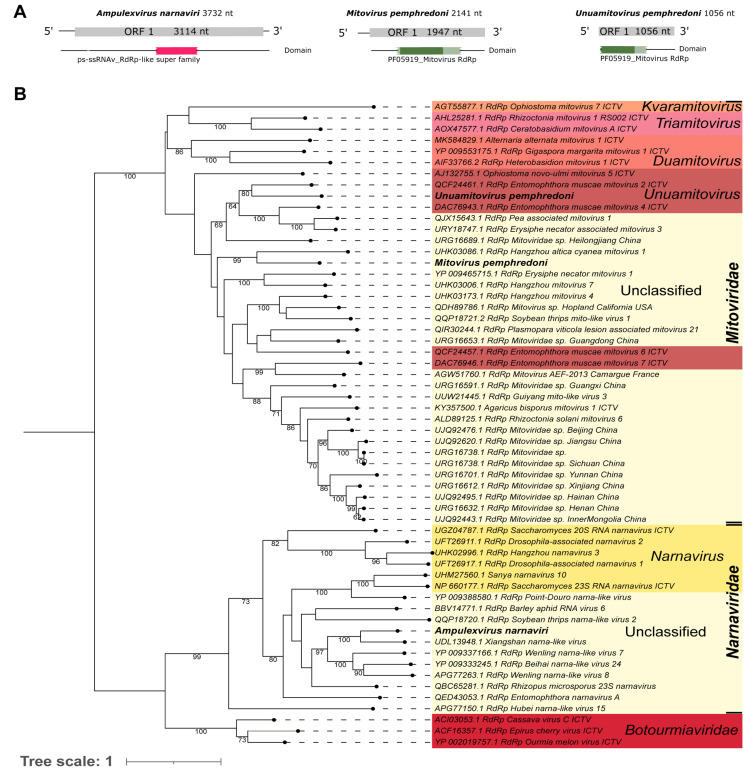
Characterization of viral sequences related to elements from the *Mitoviridae* and *Narnaviridae* families. (**A**) ORF pattern and conserved domains of *Ampulexvirus narnaviri* (**left panel**), *Mitovirus pemphredoni* (**middle panel**), and *Unuamitovirus pemphredoni* (**right panel**). (**B**) Maximum likelihood tree of elements from the *Mitoviridae* and *Narnaviridae* families. The viral genomes assembled in our work are highlighted in bold. Bootstrap values under 60 are not shown.

A library of the whole body of an adult *Ampulex compressa* (SRR14607675), from Germany, showed a viral sequence (Ac_Contig1) of 3732 nt, which presented sequence similarity at the nucleotide level (Table 1) to the Xiangshan narna-like virus hypothetical protein gene. Analysis at the protein level indicated that it is similar to a hypothetical protein of the same virus (UDL13948.1) that, despite being annotated as hypothetical, has an RdRp domain (cl40470), indicating that this protein represents the viral replicase (Table 1). Our assembled sequence has an ORF of 3114 nt|1037 aa, which is similar to its closest relative, the aforementioned *Xiangshan narna-like virus* (ORF: 3183 nt|1060 aa) (Figure 3A—left panel and Supplementary Figure S2). Based on the phylogenetic analysis, this putative virus was grouped with sequences of unclassified genera within *Narnaviridae* and was closely related to *Xiangshan narna-like virus* with 100% bootstrap (Figure 3B). Using NCBI Conserved Domain search, we were able to identify the RdRP domain cl40470 in our assembled narnavirus sequence (Figure 3A). To reflect the virus host and family, this sequence was named *Ampulexvirus narnaviri*, in accordance with recent ICTV guidelines [61].

- ***Cotesia vestalis***

We analyzed seven libraries from China of whole-body Cotesia vestalis (field adult and laboratory-reared pupae) (Supplementary Table S2). All libraries contained viral sequences showing similarity at the amino acid level to elements of three families: Virgaviridae (one putative virus, 9075 nt, detected in four libraries), Orthomyxoviridae (five segments of one putative virus, detected in three libraries), and Rhabdoviridae (one putative virus, 12,294 nt, detected in four libraries). Furthermore, three segments of a putative unclassified virus of the Bunyavirales order were identified (RdRp with 6636 nt; glycoprotein with 1579 nt; and a nucleocapsid with 868 nt). Thus, considering the presence of polymerases that lack similarity at the nucleotide level to known viruses, we detected four possible new species infecting Cotesia vestalis. 

**Figure 4:**
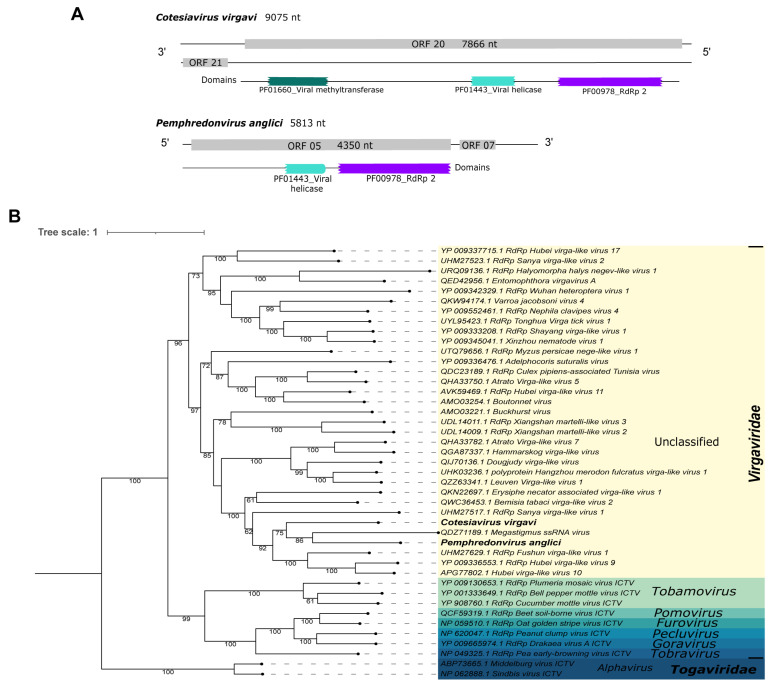
Characterization of viral sequences related to elements from the Virgaviridae family. (**A**) ORF pattern and conserved domains of Cotesiavirus virgavi (**upper panel**) and Pemphredonvirus anglici (**lower panel**). (**B**) Maximum likelihood tree of elements from the Virgaviridae family. The viral genomes assembled in our work are highlighted in bold. Bootstrap values under 60 are not shown.

First, Cv_Contig1, of 9075 nt, matched at the nucleotide level to the Abisko virus, complete genome, of 10,187 nt (Table 1). Its best hit at the amino acid level was the RdRp of Sanya virga-like virus 1 (Table 1). Due to low query coverage and identity to known viruses, this sequence likely represents a new virus that has similar ORF (7866 nt|2621 aa) and domains (PF01660_viral methyltransferase, PF01443_viral helicase, and PF00978_RdRp 2) (Figure 4A—top panel) to its best hit, the above-mentioned Sanya virga-like virus 1 (ORF 6267 nt) (Supplementary Figure S4). According to the phylogenetic analysis, this putative new virus clustered with Megastigmus ssRNA virus and Pemphredonvirus anglici, another Virgaviridae virus we describe in this work, with 75% bootstrap (Figure 4B). This new virus was named Cotesiavirus virgavi. 

**Figure 5:**
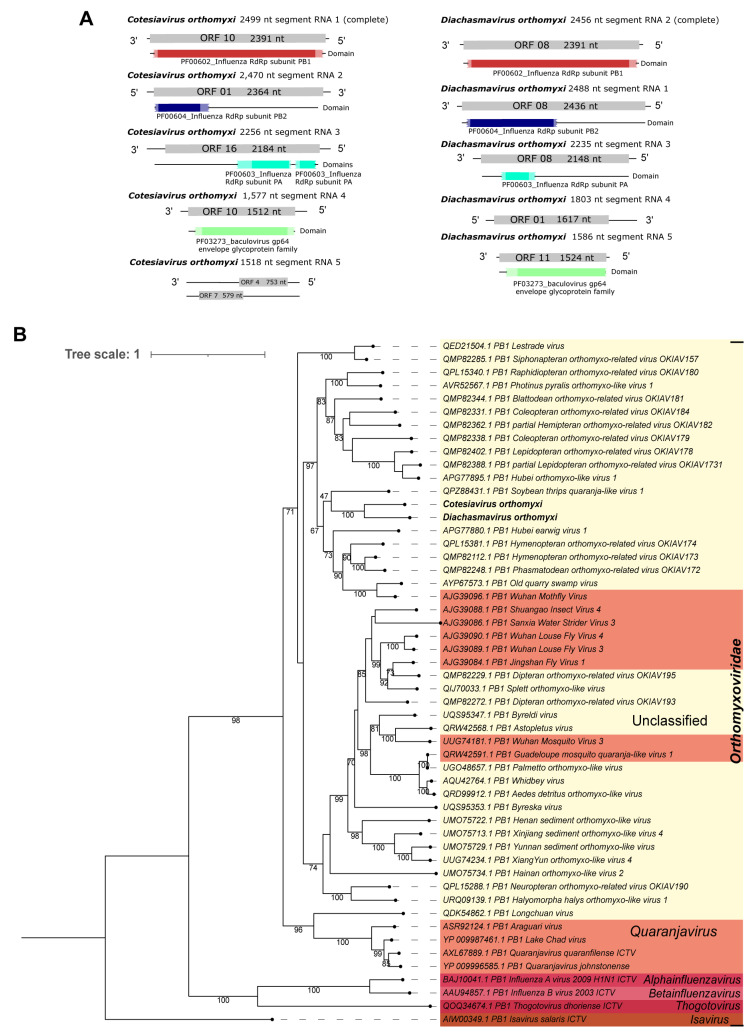
Characterization of viral sequences related to elements from the *Orthomyxoviridae* family. (**A**) Segments, ORF pattern, and conserved domains of *Cotesiavirus orthomyxi* (**left panel**) and *Diachasmavirus orthomyxi* (**right panel**). (**B**) Maximum likelihood tree of elements from the *Orthomyxoviridae* family. The viral genomes assembled in our work are highlighted in bold. Bootstrap values under 60 are not shown.

Second, five segments of an unclassified Orthomyxovirus were identified using sequence similarity search at the amino acid level as follows: Cv_RNA_segment_1 of 2499 nt matched to polymerase PB1 (Phasmatodean orthomyxo-related virus OKIAV172); Cv_RNA_segment_2 of 2470 nt was similar to polymerase PB2, partial (Phasmatodean orthomyxo-related virus OKIAV172); Cv_RNA_segment_3 of 2256 nt matched to polymerase PA, partial (Hymenopteran orthomyxo-related virus OKIAV171); Cv_RNA_segment_4 of 1577 nt presented similarity to hemagglutinin (Hymenopteran orthomyxo-related virus OKIAV173); and Cv_RNA_segment_5 of 1518 nt matched to the nucleocapsid protein (Blattodean orthomyxo-related virus OKIAV181) (Table 1). Four segments have the expected sizes and ORFs, according to close orthomyxoviruses (Supplementary Figure S4). Furthermore, these segments have domains that match to those identified in the closely related above-mentioned orthomyxoviruses (PF00602_Influenza RdRp subunit PB1, PF00604_Influenza RdRp subunit PB2, PF00603_Influenza RdRp subunit PA, and the PF03273_baculovirus gp64 envelope glycoprotein family), as illustrated in Figure 5A—left panel. Because PB1 is the polymerase subunit most conserved among orthomyxoviruses [62], and it has been used in other studies [63,64], we selected this segment to perform the phylogenetic analysis of the orthomyxoviruses identified. Cv_RNA_segment_1 (PB1 RdRp) clustered with Diachasmavirus orthomyxi (Figure 5B), another orthomyxovirus we describe in this work, with 100% bootstrap (Figure 5A,B—right panel). Also, they clustered with sequences of unclassified genera in the Orthomyxoviridae family. To indicate the original host and viral family, this new virus was named Cotesiavirus orthomyxi.

**Figure 6:**
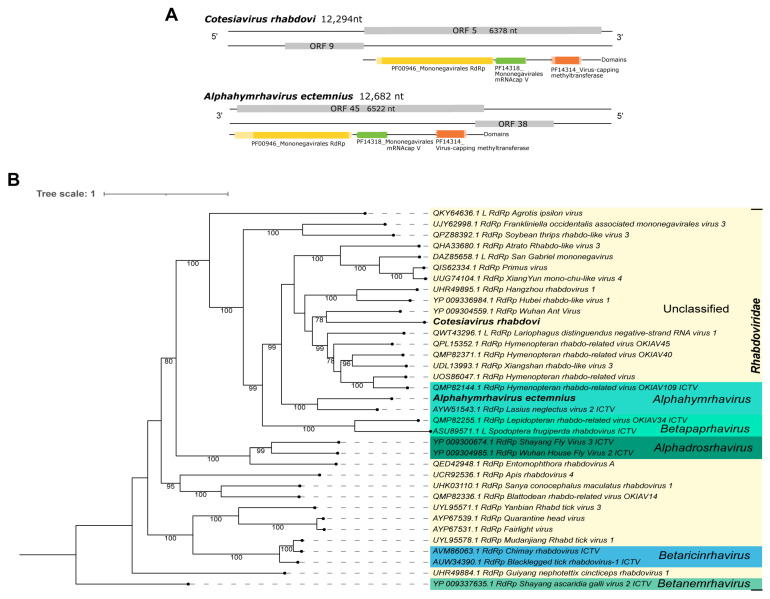
Characterization of viral sequences related to elements from the *Rhabdoviridae* family. (**A**) ORF pattern and conserved domains of *Cotesiavirus rhabdovi* (**upper panel**) and *Alphahymrhavirus ectemnius* (**lower panel**). (**B**) Maximum likelihood tree of elements from the *Rhabdoviridae* family. The viral genomes assembled in our work are highlighted in bold. Bootstrap values under 60 are not shown.

Third, Cv_Contig2, of 12,294 nt, presented limited similarity at the nucleotide level (Table 1) to *San Gabriel mononegavirus,* of 12,620 nt. Its two best hits at the amino acid level were the RdRp (*Hymenopteran rhabdo-related virus*) and RdRp *Wuhan Ant virus* (Table 1). This new virus also has the longest ORF (6378 nt|2125 aa) and domains (PF00946_Mononegavirales RdRp, PF14318_Mononegavirales mRNA-cap V, and PF14314_virus-capping methyltransferase) (Figure 6A—top panel) according to the expected ORF of its best hit, *Wuhan ant virus* (ORF 6357 nt) (Supplementary Figure S4). Based on the phylogenetic analysis, this virus clustered with sequences of unclassified genera of *Rhabdoviridae* family and was closely related to *Wuhan ant virus* with 78% bootstrap (Figure 6B). This new virus was named *Cotesiavirus rhabdovi*, reflecting the host and viral family.

**Figure 7:**
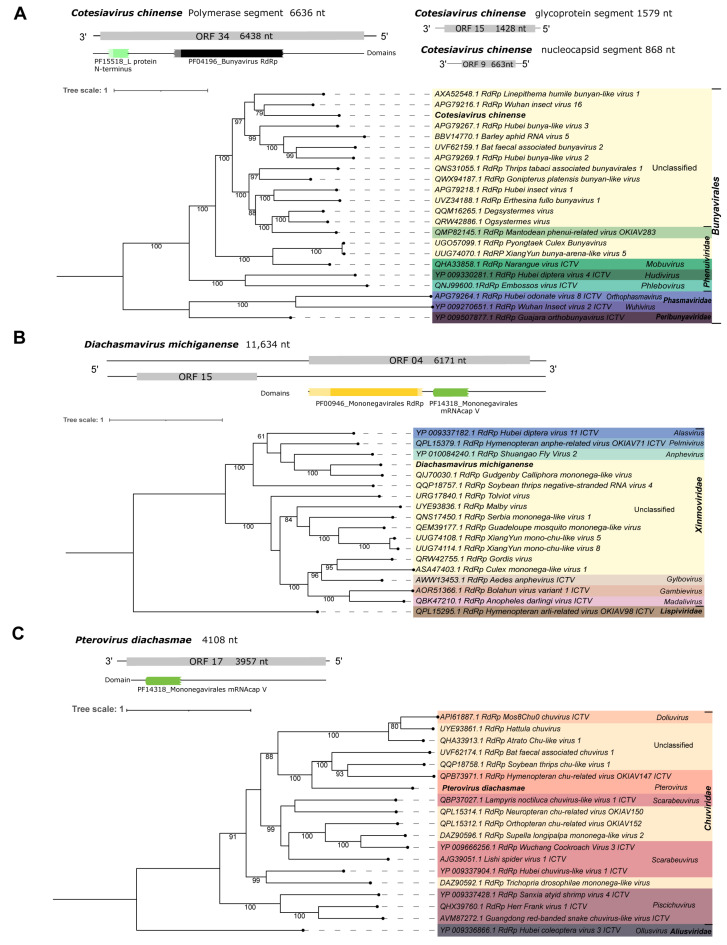
Characterization of viral sequences related to elements from the *Bunyavirales, Xinmoviridae*, and *Chuviridae* families. (**A**) Segments, ORF pattern, conserved domains, and maximum likelihood tree of *Cotesiavirus chinense.* (**B**) ORF pattern, conserved domains, and maximum likelihood tree of *Diachasmavirus michiganense.* (**C**) ORF pattern, conserved domain, and maximum likelihood tree of *Pterovirus diachasmae.* The viral genomes assembled in our work are highlighted in bold. Bootstrap values under 60 are not shown.

Fourth, Cv_Contig3, of 6636 nt in length, is one of the three sequences that shows similarity to elements from the *Bunyaviridae* family. The best hit of Cv_Contig3 at the nucleotide level was the Wuhan insect virus 16 RdRp gene. Also, at the amino acid level, it matched with the polymerase of the same virus (Table 1). Its longest ORF (6438 nt|2145 aa) is as expected based on its closest virus, *Wuhan insect virus 16* (ORF 6417 nt). In addition, there are two domains (PF15518_L-protein N-terminus and PF04196_Bunyavirus RdRp) that reinforce its classification as a member of *Bunyavirales* (Figure 7A). The sequence showing similarity to a glycoprotein, of 1579 nt, matched with *Hymenopteran phenui-related virus OKIAV282* while the sequence identified as nucleoprotein, of 868 nt, was closely related to *Hymenopteran phenui-related virus OKIAV275* (Table 1). They are represented in Figure 7A but did not show any conserved domain similar to the closest viral sequences (Supplementary Figure S4). Based on the phylogenetic analysis using the polymerase segment, this putative new virus is related to unclassified elements from the *Bunyavirales* order. Its closest virus was *Wuhan insect virus 16* with 79% bootstrap (Figure 7A). This new virus was named *Cotesiavirus chinense.*

- ***Diachasma alloeum***

Two libraries of adult whole-body *Diachasma alloeum* from the United States revealed new viruses classified into at least three families: *Xinmoviridae* (one putative virus, 11,634 nt, repeated in two libraries), *Orthomyxoviridae* (five segments of one putative virus, repeated in two libraries), and *Chuviridae* (one putative virus, 4108 nt). 

First, Da_Contig1, of 11,634 nt, presented similarity at the nucleotide level to *Gudgenby Calliphora mononega-like virus*, 12,763 nt (Table 1). Its best hit at the amino acid level was the RdRp of the same virus (Table 1). Due to low similarity, it is probably a new virus with the longest ORF (6171 nt|2056 aa) and domains (PF00946_Mononegavirales RdRp and PF14318_Mononegavirales mRNA-cap V) as expected through comparison with its closest aforementioned virus (Figure 7B and Supplementary Figure S4). The phylogenetic analysis pointed to this virus as an unclassified member of the *Xinmoviridae* family and the *Mononegavirales* order. Also, this virus clustered with *Gudgenby Calliphora mononega-like virus* with 100% bootstrap (Figure 7B). This putative new virus was named *Diachasmavirus michiganense* to describe its original host and geographical origin. 

Second, a putative new orthomyxovirus had five segments identified through sequence similarity at the amino acid level as follows: Da_RNA_segment_2 of 2456 nt matched polymerase PB1, *Hymenopteran orthomyxo-related virus OKIAV173*. Da_RNA_segment_1, of 2488 nt, matched polymerase PB2, *Phasmatodean orthomyxo-related virus OKIAV172*; Da_RNA_segment_3, of 2235 nt, matched polymerase PA, *Hymenopteran orthomyxo-related virus OKIAV173*; Da_RNA_segment_4, of 1803 nt, matched putative nucleocapsid, *Old quarry swamp virus*; and Da_RNA_segment_5, of 1586 nt, matched hemagglutinin, *Hymenopteran orthomyxo-related virus OKIAV173* (Table 1). The five segments presented expected sizes and domains (PF00602_Influenza RdRp subunit PB1, PF00604_Influenza RdRp subunit PB2, PF00603_Influenza RdRp subunit PA, and the PF03273_baculovirus gp64 envelope glycoprotein family) similar to their closest orthomyxoviruses (Figure 5A—right panel and Supplementary Figure S4). Based on the phylogenetic analysis of the PB1 segment, this virus clustered with sequences of an unclassified genus of the *Orthomyxoviridae* family. Also, its closest related virus was the *Cotesiavirus orthomyxi* already described in this work (100% bootstrap) (Figure 5B). Together, the two viruses clustered with *Soybean thrips quaranja-like virus 1*, although it occurred with low bootstrap (47%). This putative new virus was named *Diachasmavirus orthomyxi* based on its original host and viral family. It is worth noting that, we compared the two PB1 segments of *Cotesiavirus orthomyxi* and *Diachasmavirus orthomyxi* (22% query cover; e-value = 3 × 10^−24^; 290/446 (65%) id) using BlastN, and these sequences are unlikely from the same virus. Although, they seem to be phylogenetically close, occupying the same clade in the phylogenetic tree (Figure 5B). This may occur either due to the lower sampling of wasp viruses or because they are substantially divergent from the other viruses in the tree (Figure 5B). 

Third, Da_Contig2, of 4108 nt, presented similarity at the amino acid level to the RdRp of *Hymenopteran chu-related virus OKIAV147* (Table 1). Based on the phylogenetic analysis, this virus clustered with 100% bootstrap with the same virus, classified as *Pterovirus*, and two others unclassified at the genus level. This is a putative new *Pterovirus* due to the low similarity of the sequence, even though it is incomplete. The longest ORF should be 6606 nt, with Mononegavirales RdRp and mRNA capping domains, according to the best hit mentioned above (Figure 7C and Supplementary Figure S4). Here, we obtained a smaller ORF (3957 nt|1318 aa) and just the PF14318_Mononegavirales mRNA cap V domain for this viral fragment. Still, the phylogenetic tree characterized it as being from *Pterovirus* genus of the *Chuviridae* family (Figure 7C). To indicate the viral genus and original host, this new virus was named *Pterovirus diachasmae.*

- ***Ectemnius lituratus***

For the single library of *Ectemnius lituratus’* head and thorax (ERR6054901) included in this study, from the United Kingdom, we detected El_Contig1. This is a 12,682 nt-long sequence that showed similarity at the nucleotide level to *Hymenopteran rhabdo-related virus OKIAV38*, 12,376 nt. Also, it was identified at the amino acid level as the RdRp of *Lasius neglectus virus 2* (Table 1). It has low similarity with the above-mentioned sequences, but the longest ORF (6522 nt|2173 aa) and domains (PF00946_Mononegavirales RdRp, PF14318_Mononegavirales mRNAcap V, and PF14314_virus-capping methyltransferase), illustrated in Figure 6A—bottom panel, which are concordant with its closest related virus, *Lasius neglectus virus 2* (Supplementary Figure S4). Based on the phylogenetic analysis, this virus clustered with sequences of the *Alphahymrhavirus* genus of *Rhabdoviridae*. Its closest virus relative is *Lasius neglectus virus 2,* with 100% bootstrap (Figure 6B). We suggest that this is a new complete virus, named *Alphahymrhavirus ectemnius* due to its viral genus and original host.

- ***Pemphredon lugubris***

The single library of the abdomen of an adult *Pemphredon lugubris* (ERR8571638), from the United Kingdom, revealed the largest number of new viruses for a wasp species. We detected seven putative new viruses and one known mitovirus infecting *P. lugubris*. The viruses were distributed among at least four families: *Virgaviridae* (one putative new virus sequence), *Partitiviridae* (one new virus sequence), *Endornaviridae* (three putative new virus sequences), and *Mitoviridae* (two new virus sequences and one known virus sequence). 

Pl_Contig1, of 5813 nt, presented similarity at the amino acid level to the hypothetical protein of *Megastigmus ssRNA virus* (Table 1). Its longest ORF has 4350 nt|1449 aa long and two domains (PF01443_viral helicase and PF00978_RdRp 2) (Figure 4A—bottom panel). This viral sequence is incomplete since its closest virus mentioned previously has a genome of 12,061 nt with two ORFs (2985 nt and 4980 nt) and four domains (PF01660_viral methyltransferase and PF01728_FtsJ-like methyltransferase; PF01443_viral helicase and PF00978_RdRp 2, respectively) (Supplementary Figure S4). The phylogenetic analysis classified this viral sequence as a member of the *Virgaviridae* family and it clustered with sequences of unclassified genera. It was closely related to *Megastigmus ssRNA virus*, with 86% bootstrap (Figure 4B). This virus was named *Pemphredonvirus anglici* to indicate its original host and geographical origin.

**Figure 8:**
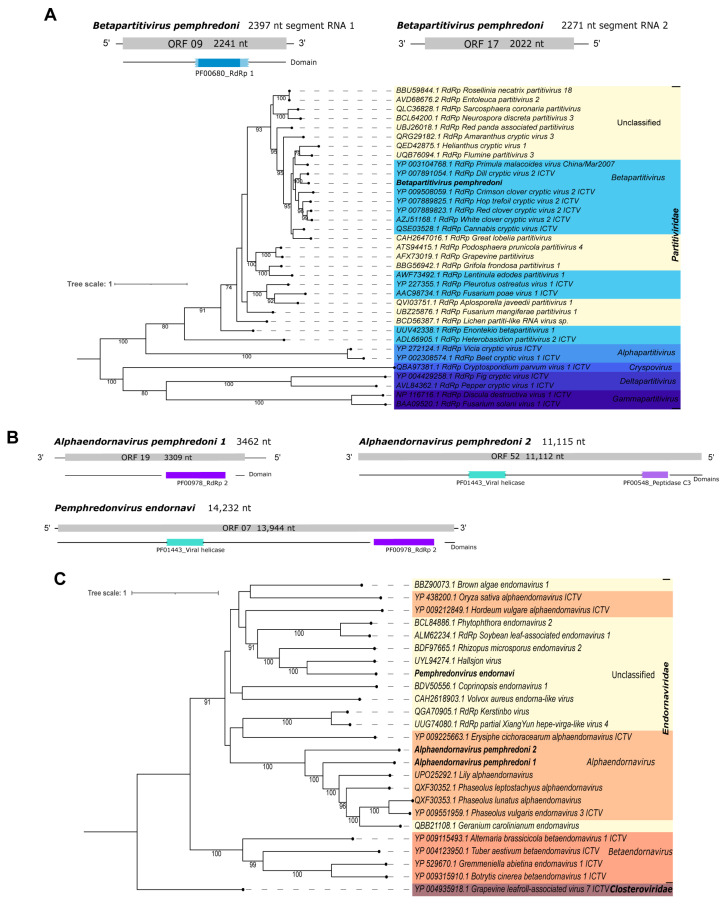
Characterization of viral sequences related to elements from the *Partitiviridae* and *Endornaviridae* families. (**A**) Segments, ORF pattern, conserved domain, and maximum likelihood tree of *Betapartitivirus pemphredoni.* (**B**) ORF pattern and conserved domains of *Alphaendornavirus pemphredoni* 1 (**upper left panel**), *Alphaendornavirus pemphredoni 2* (**upper right panel**), and *Pemphredonvirus endornavi* (**lower panel**). (**C**) Maximum likelihood tree of elements from the *Endornaviridae* family. The viral genomes assembled in our work are highlighted in bold. Bootstrap values under 60 are not shown.

Pl_Contig2, of 2397 nt, is a putative two-segmented new virus identified by sequence similarity at the nucleotide level as *Dill cryptic virus 2* isolate IPP_hortorum segment RNA 1, complete sequence (Table 1). It has the longest ORF (2241 nt|746 aa) with a PF00680_RdRp 1 domain. The second segment of this virus (Pl_Contig2.2) is 2271 nt long and was also identified at the nucleotide level as *Dill cryptic virus 2* isolate IPP_hortorum segment RNA 2, complete sequence (Table 1), with the longest ORF of 2022 nt in length, without domains (Figure 8A– bottom panel). The ORFs and RdRp domain we detected are concordant with those expected for the closest virus identified at the nucleotide level (Supplementary Figure S4). The phylogenetic analysis classified this virus as a member of the *Betapartitivirus* genus within the *Partitiviridae* family. This virus also clustered with *Dill cryptic virus 2* with 100% bootstrap (Figure 8A). It was named *Betapartitivirus pemphredoni*. 

Pl_Contig3, of 3462 nt, is a putative new virus identified by the similarity of its sequence, at the nucleotide level, to *Phaseolus lunatus alphaendornavirus* isolate SN35. Also, it matched at the amino acid level to *Lily alphaendornavirus* (Table 1). This is an incomplete viral sequence because its closest virus is 16,483 nt long. Despite this, this sequence has an ORF (3309 nt|1102 aa) that contains a PF00978_RdRp 2 domain (Figure 8B—left panel), as expected for viruses of this group (Figure 8B and Supplementary Figure S4). The phylogenetic analysis classified this virus as a member of the *Alphaendornavirus* genus of the *Endornaviridae* family. Also, this virus clustered with other sequences of the same genus with 100% bootstrap (Figure 8C). To indicate its viral genus and original host, this virus was named *Alphaendornavirus pemphredoni 1*. 

Pl_Contig4, of 11,115 nt, did not present any related sequence at the nucleotide level. This putative new virus showed similarity at the amino acid level to Geranium carolinianum endornavirus polyprotein (Table 1). The longest ORF of this virus is 11,112 nt|3703 aa, and it contains two domains (PF01443_viral helicase and PF00548_peptidase C3) (Figure 8B—right panel). The viral sequence Pl_Contig4 is probably incomplete since the closest virus identified at the amino acid level is 14,625 nt long. However, the domains are concordant with endornaviruses (Figure 8B and Supplementary Figure S4). According to the phylogenetic analysis, this virus was classified as *Alphaendornavirus* within *Endornaviridae* (Figure 8B). This sequence clustered with other alphaendornaviruses including its closest hit, *Alphaendornavirus pemphredoni 1*, described above in this work, with 100% bootstrap (Figure 8B,C). The comparison of the two Alphaendornaviruses of *P. lugubris* at the nucleotide level did no show significant sequence similarity, suggesting that they are distinct viruses. This virus was named *Alphaendornavirus pemphredoni 2*. 

Pl_Contig5, of 14,232 nt, had the best hit at the amino acid level with Hallsjon virus putative polyprotein (Table 1). Its longest ORF is 13,944 nt|4647 aa long, and it has two domains (PF01443_viral helicase and PF00978_RdRp 2) (Figure 8B—bottom panel). The sizes of the genome and the longest ORF are as expected for endornaviruses like *Hallsjon virus* (Supplementary Figure S4). However, in the phylogenetic tree, this putative new virus is an unclassified *Endornaviridae* member because it clustered with sequences of unclassified genera of this family (Figure 8C). In addition, this virus clustered with *Hallsjon virus* with 100% bootstrap. To mention its original host and viral family, this putative new virus was named *Pemphredonvirus endornavi*. What is noteworthy is that *Pemphredonvirus endornavi* has no similarity at the nucleotide level to *Alphaendornavirus pemphredoni 1*, and it had a very small hit with *Alphaendornavirus pemphredoni 2* (0% query cover; e-value = 0.047; 23/27(85%) id). Therefore, we suggest that they are three distinct endornaviruses infecting the same wasp species. 

Pl_Contig6, of 2141 nt, did not presented hits at the nucleotide level. Its best hit was at the amino acid level with the RdRp of *Hangzhou altica cyanea mitovirus 1* (Table 1). Its longest ORF, translated with genetic code 4, is 1947 nt|648 aa long. Also, this ORF has a PF05919_Mitovirus RdRp domain (Figure 3A—middle panel). As expected for similar mitoviruses, like *Hangzhou mitovirus 4* (its second hit in BlastX), the genome and ORF sizes agree for this group of viruses (Supplementary Figure S4). Thus, due to low similarity with the already described mitoviruses and based on the phylogenetic analysis, we suggest that this is a new mitovirus species. It clustered with sequences of unclassified genera of the *Mitoviridae* family and its closest virus was *Hangzhou altica cyanea mitovirus 1*, with 99% bootstrap (Figure 3B). To reflect the viral family and host, this putative new virus was named *Mitovirus pemphredoni.*

Pl_Contig7, of 1056 nt, did not present similarity at the nucleotide level with known viruses. Its best hit at the amino acid level was the RdRp of *Entomophthora muscae mitovirus 2* (Table 1). Its longest ORF, translated with genetic code 4, is 1056 nt|352 aa long and it has a partial PF05919_Mitovirus RdRp domain (Figure 3A—right panel). Its closest virus, *Entomophthora muscae mitovirus 2,* has an ORF of 2070 nt in length and a complete domain PF05919_Mitovirus RdRp (Supplementary Figure S4). Based on the phylogenetic analysis, this putative virus clustered with sequences from the genus *Unuamitovirus* (*Mitoviridae* family) and was closely related to *Entomophthora muscae mitovirus 2,* with 80% bootstrap (Figure 3B). To indicate its viral genus and original host, this virus was named *Unuamitovirus pemphredoni.*

The comparison of *Mitovirus pemphredoni 1* (2141 nt) and *Unuamitovirus pemphredoni* (1056 nt) showed very limited similarity at the nucleotide level with only a fraction of the sequences with similarity to each other (2% query cover; e-value = 0.010; 36/50 (72%) id). Thus, we suggest that they are two distinct species of novel mitoviruses infecting the same wasp species (*P. lugubris*). 

Finally, Pl_Contig10, the known mitovirus of *P. lugubris* is a sequence of 1143 nt long and was identified at the nucleotide level as *Hubei narna-like virus 25* strain SCM51430 of 2375 nt (100% query cover; e-value = 0.0; 1054/1144 (92%) id) (Table 1 and Supplementary Figure S4). The assembled sequence is likely incomplete, but it presented an ORF translated by the genetic code 4 (888 nt|295 aa), which contains a PF05919_Mitovirus RdRp domain similar to the reference virus. This virus was firstly isolated from a Dipteran host in China [65].

- ***Telenomus podisi***

The *Telenomus podisi’s* whole-body libraries from Brazil [66] that we included in this study did not reveal new viral sequences. On the other hand, we assembled Tp_Contig1, a sequence of 8285 nt with sequence similarity to *Halyomorpha halys virus* isolate Beltsville, complete genome, of 9263 nt, at the nucleotide level (100% query cover; e-value = 0.0; 7351/7518 (98%) id) (Table 1 and Supplementary Figure S4). This iflavirus was isolated for the first time in 2013 from the brown marmorated stink bug, *Halyomorpha halys,* at Beltsville, MD, USA [67]. *Telenomus podisi* is a natural enemy of stink bug species (Hemiptera: *Pentatomidae*) and also occurs at Maryland [23]. Therefore, the identification of the same virus in prey and predator suggest this may be a case of virus transmission from ‘prey to predator’ or vice-versa.

### 3.3. Quantification of Viral Transcripts

**Figure 9:**
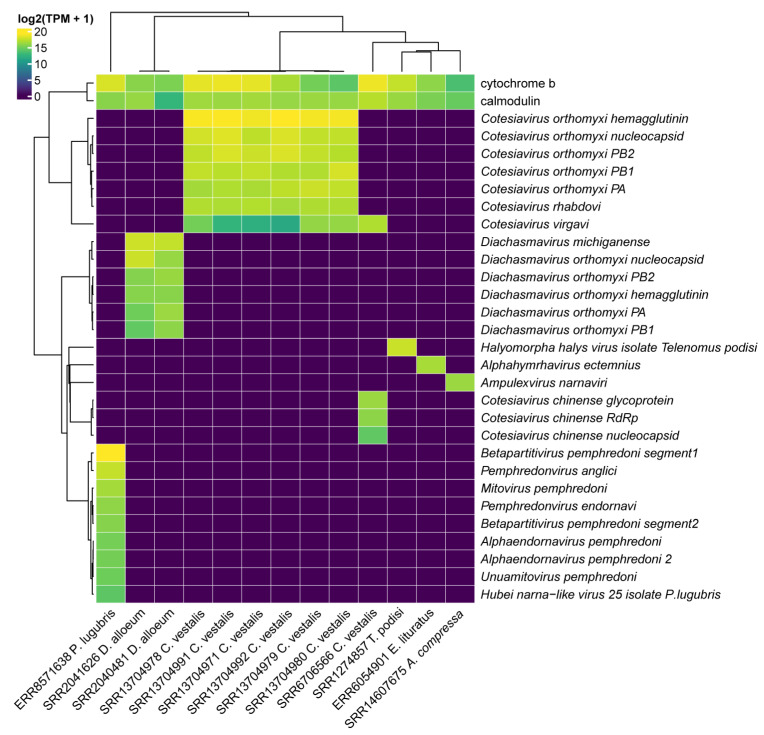
Assessment of the transcriptional activity of viral sequences identified in parasitoid wasps. Abundance was estimated in transcripts per million (TPM). The viral segments are shown in rows while the RNA deep sequencing libraries and its respective wasp origin are indicated in the columns. The abundance of the constitutive genes (cytochrome b and calmodulin) are shown for each of the parasitoid wasp species.

In order to quantify the transcriptional activity of the virus identified in each species, we compared the raw reads from each library against viral genomes and the constitutive markers genes *cytochrome b* and *calmodulin*. The transcriptional activity of both constitutive genes was detected for all wasp libraries (Figure 9). 

As expected, for each parasitoid species, there is a distinct pattern of virome since different viruses were detected. The differences are likely due to the species origin. Indeed, libraries of laboratory-reared *C. vestalis* differ in their viruses’ composition from field-collected wasp specimens. In the first case, six libraries had *Cotesiavirus rhabdovi* (2536–4679 TPM), and Cotesiavirus orthomyxi segments (1712–10,800 TPM) were the most abundant viral transcripts (Figure 9). On the other hand, we observed *Cotesiavirus chinense* exclusively in field-collected *C. vestalis* samples; *Cotesiavirus virgavi* was detected in samples derived from both origins. 

*Diachasmavirus michiganense* (15,235 TPM) was the most abundant virus for *D. alloeum* wasps, followed by Diachasmavirus orthomyxi segments, while *Pterovirus diachasmae* presented the lowest abundance. *Alphahymrhavirus ectemnius* from *E. lituratus* had 2902 TPM. Regarding *P. lugubris*, the most abundant virus was Betapartitivirus pemphredoni segment1 (38,302 TPM), followed by *Pemphredonvirus anglici* (7327 TPM) and *Mitovirus pemphredoni* (2900 TPM). Finally, *Halyomorpha halys virus* isolate Telenomus podisi had 5438 TPM. 

Assessment of the transcriptional activity of the viral segments indicated consistency in the abundance of segments from the same viral species, such as those identified in *P. lugubris* (*Betapartitivirus pemphredoni*), *C. vestalis* (*Cotesiavirus orthomyxi*), and *D. alloeum* (*Diachasmavirus orthomyxi)*, which contained viruses with segmented genomes (Figure 9).

## 4. Discussion

On the evolutionary history of the parasitoids, they diffused among a richness of hosts and parasitoid ecological niches, such as egg-parasitoidism, hyperparasitoidism, kleptoparasitoidism, and polyembryony [68]. On many occasions, this was possible due to the cooptation of the viruses to subjugate their hosts [68]. In addition, the probability of parasitoids expanding their geographical distribution depends on factors such as suitable host species and propitious environmental conditions (climate, host plants, host habitat, etc.) [69]. To illustrate, climate change may influence predator–prey relationships by modifying the behavior or distribution of the species involved [70]. Due to the wide geographical distribution of the selected parasitoid species (Figure 1), for instance, it is possible for wasp viruses to spread to other beneficial insects, such as pollinators [71,72]. Of equal importance, current RNA viruses circulating in ecologically important parasitoid wasps may synergistically affect the ecological interactions [73,74] of parasitoid hosts, biodiversity, ecosystem services dispensed, and the safety of using such wasps as biocontrol agents. Additionally, non-pathogenic viruses may establish mutualistic interactions with their hosts [75]. For those reasons, it is important to know which viruses compose the microbiome of these highly diverse ecosystem service providers. Consequently, this knowledge may help to mitigate the loss of biodiversity and unwatched viral spillover. 

**Figure 10:**
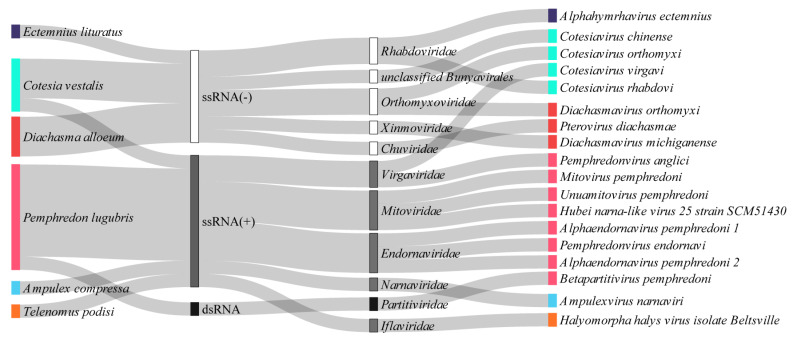
Baltimore and viral family classification of the RNA viral species identified in the parasitoid wasps. Each viral sequence is represented with the same color of its source host. The viral diversity is represented by Baltimore classification: ssRNA(−) white bar, ssRNA(+) gray bar, and dsRNA black bar. Viral families are represented next to their Baltimore categories.

Here, we found 18 viruses that could be classified into 10 families (*Iflaviridae, Endornaviridae, Mitoviridae, Partitiviridae, Virgaviridae, Rhabdoviridae, Chuviridae, Orthomyxoviridae, Xinmoviridae,* and *Narnaviridae*) and in the *Bunyavirales* order (Figure 10). Sixteen of them likely represent novel viral species. 

Viruses of *Iflaviridae* are non-enveloped with monopartite, positive-stranded RNA genomes of 9–11 kb, and infections can be asymptomatic or symptomatic (behavioral changes, premature mortality, and malformations) [76]. They have been found in Arthropoda hosts, such as honey bees, wasps, *Varroa destructor* [77], and are transmitted mainly by the ingestion of contaminated food [76]. In this study, we found a previously described iflavirus *Halyomorpha halys virus* (TP_Contig1) in *Telenomus podisi* libraries from Brasília, Brazil; the original study published in 2015 contains important details to elucidate whether the virus is circulating in the parasitoid wasp. It is interesting that the study was conducted with three different species of stink bugs because in Brazil the major pests of Pentatomidae are *Euchistus heros*, *Chinavia ubica*, and *Dichelops melacanthus*, with them principally attacking soybean crops [66]. Moreover, in 2019, another study was published reporting an iflavirus of four species of stink bugs, and it was conducted with the same three pest species and libraries already published in 2015 [66]. Therefore, they reported the same *Halyomorpha halys virus* species asymptomatically circulating in the original host and the other three South American stink bugs [78]. Here, we detected the same virus in the parasitoid of those *Pentatomidae* species. It is possible that the virus passed from the prey to the parasitoid during its development in the laboratory and remained active after the adult’s eclosion. What is noteworthy is that the adult wasps obtained from laboratory colonies were maintained separated from their prey and nourished with pure honey and, for reproduction, they received *E. heros* eggs to parasitize [66]. Since 20-day-old wasps were used for RNA extraction, it is unlikely that the virus originated from *E. heros*’ egg remnants. Therefore, the prey’s virus passed to its parasitoid wasp previously and is probably circulating in *T. podisi* as well. 

Three new putative viruses of *Endornaviridae* (*Alphaendornavirus pemphredoni 1*, *Pemphredonvirus endornavi*, and *Alphaendornavirus pemphredoni 2*) were identified in *P. lugubris*. As stated by the ICTV, Endornaviruses are positive-sense, single-stranded RNA viruses with genomes of 9.7 to 17.6 kb. They have been found in fungi, oomycetes, and plants [79]. However, recently, two Alphaendornaviruses, a strain of *Hallsjon virus*, firstly described in *Culex torrentium* [80], and a novel virus named *Tvarminne alphaendornavirus* [81] were detected in mosquitoes from Finland. Here, *Pemphredonvirus endornavi* had sequence similarity at the amino acid level to *Hallsjon virus*, but with low identity. 

Pl_Contig10, one previously described mitovirus, and two new putative mitoviruses (*Mitovirus pemphredoni* and *Unuamitovirus pemphredoni*) were detected in *P. lugubris*. *Mitoviridae* viruses are RNA viruses with positive-sense, single-stranded, adenine–uracil (AU) rich genomes. These are capsidless virusesand their genomes range from 2151 to 4955 nt, with one ORF coding an RdRp domain that ranges from 636 to 1137 aa [82]. Thus far, several putative mitoviruses have been described from a diversity of fungi and Plantae species [83,84], and a recently published study found evidence to enlarge the host possibilities beyond them. Based on phylogenetic reconstruction, Jacquat and cols (2022) suggest the existence of a lineage of mitoviruses derived from animals because the putative mitoviruses do not cluster with species of fungal origin [82]. *Mitovirus pemphredoni* and *Unuamitovirus pemphredoni* clustered with other sequences from several sequences from fungus and plant viruses (Figure 3). Thus, it is not possible to ensure whether they are replicating inside the wasp’s mitochondria or are derived from an external source. through contamination. Interestingly, *Unuamitovirus pemphredoni* clustered with 80% bootstrap with *Entomophthora muscae mitovirus 2* (Figure 3B), a virus from an entomopathogenic fungus that infects, manipulates, and kills its Dipteran hosts to favor its dispersal through spores [85]. *Entomophthoraceae* appeared in our metagenomics analysis (Figure 2), pointing out that *P. lugubris* may be a new host for it. What is noteworthy is that the fungi of this family have already been detected in aphid pests in Argentina [86], the main prey of this parasitoid wasp. More studies are needed to elucidate if such mitoviruses contribute somehow to *Entomophthora muscae’s* successful parasitoid strategy. Equally, further analysis should be performed to confirm the relationship between *Entomophthoraceae* species and *P. lugubris*. 

Another family related to mitoviruses is the *Narnaviridae* family, which contains non-encapsidated positive-stranded RNA viruses that range from 2.3 to 3.6 kb [87]. We found in our analysis that *Ampulexvirus narnaviri* clustered with *Xiangshan narna-like virus*, which was obtained from a mixed pool of several species of insects [88]. It was not possible to classify this species at the genera level since all of the closest species are also defined as unclassified elements within the *Narnaviridae* family (Figure 3B). 

*P. lugubris* also had two segments of a *Betapartitivirus* of the *Partitiviridae*. As maintained by the ICTV, this genus has viruses from plants or fungi. Partitivirus are small and non-enveloped with two segments of double-stranded RNA genomes that range from 3 to 4.8 kb [89]. Metagenomic studies have shown that partitiviruses are also common in insects such as flies and mosquitoes [80,90]. In addition, a Partiti-like virus was found in honey bees of several apiaries across the USA, probably causing a mild or asymptomatic infection [91]. 

*Virgaviridae* viruses have a positive-sense, single-stranded RNA genome of 6.3 to 13 kb in length. There are non-segmented and segmented members within this family. They are non-enveloped viruses present in plants [92]. However, studies have related their occurrence in insects [65,93]. Here, we found two members of unclassified genera from *Virgaviridae*. Interestingly, *Cotesiavirus virgavi* and *Pemphredonvirus anglici* clustered with *Megastigmus ssRNA virus* (Figure 4), a virus isolated from *Megastigmus spermotrophus* (Hymenoptera: Torymidae), a North-American seed parasitoid wasp present in Europe [94]. 

*Rhabdoviridae* members are viruses with negative-sense RNA genomes of 10 to 16 kb. As stated by the ICTV, they can infect a wide range of hosts, including vertebrate animals, plants, and arthropods. Further, several rhabdoviruses are transmitted by arthropods and may be pathogenic to humans, livestock, fish, and farm crops [95]. In this study, two novel rhabdoviruses were identified. *Cotesiavirus rhabdovi* clustered with *Wuhan Ant Virus*, firstly described in China [3], and isolated from *Camponotus japonicus*, an ant native to eastern Asia. The *C. vestalis’* libraries we have studied are from the same country. Similarly, *Alphahymrhavirus ectemnius* clustered with a virus of an ant host, *Lasius neglectus virus 2*, isolated from *Lasius neglectus* sampled from Cambridge, UK, once again the same country of the library’s origin [96]. 

*Chuviridae* is a latterly described family of negative-sense single-stranded RNA viruses that infect various arthropods, such as mosquitoes [65]. Here, we detected *Pterovirus diachasmae* that clustered with three other viral sequences from: *Aphelinus abdominalis* (*Hymenopteran chu-related virus OKIAV147),* soybean thrips or *Neohydatothrips variabilis* (*Soybean thrips chu-like virus 1*), and a bat (*Bat faecal associated chuvirus 1). Aphelinus abdominalis* is an aphid parasitoid wasp used for the biocontrol of lettuce crops [97]. Soybean thrips are worrying agricultural pests and also vectors of diverse plants’ viruses [98]. 

According to the ICTV, *Orthomyxoviridae* and *Xinmoviridae* are families of negative-sense single-stranded RNA viruses that have been found in insects [81,99]. In particular, orthomyxoviruses are constituted by 6 to 8 segments and have been isolated from insect pollinators in China and Korea [88,100]. Here, we detected two putative orthomyxoviruses composed by five segments (*Cotesiavirus orthomyxi* and *Diachasmavirus orthomyxi*) that clustered together. Regarding xinmoviruses, we described *Diachasmavirus michiganense,* a putative *Xinmoviridae* virus that clustered with *Gudgenby Calliphora mononega-like virus*, which was isolated from ectoparasites (blowflies) of rabbits [101].

Viruses of the *Bunyavirales* order have been described in many hosts (such as arthropods, plants, and mammals), since this is the most abundant RNA virus order with eight families [102]. In this study, we found a member of this order, named *Cotesiavirus chinense*, which clustered with *Wuhan insect virus 16*. 

More studies on the virome of parasitoid wasps are needed to clarify the prevalence of the new viruses identified here at the population or species level. Identifying which viruses are present in the prey may indicate a possible viral origin. Further investigations looking for viruses in RNA-seq from different species, locations, tissues, and life stages of insects, whether predators or prey, will indicate whether the viruses establish infection or are only obtained mechanically or by contamination, without replication in more than one host. As there are still few studies covering this theme, it is not possible to discuss the scope of these viruses in depth. However, simply increasing the number of viral sequences described from parasitoid wasps will certainly facilitate the identification and classification of viral agents, favoring the development of more applied studies on this subject in the future.

To sum up, parasitoid wasps are the best adapted insects and are highly diverse, with more than 50% of all known Hymenopteran species classified within this group [68]. Furthermore, there is a pressing need for new methods of biological control to reduce pesticides’ use. In conclusion, a great diversity of associated viruses was registered among the parasitoid wasps analyzed in this study. Therefore, parasitoids and their viruses should be part of the research focus for the following reasons: maximizing the use of parasitoid wasps to control agricultural pests; applying wasps to fields without putting the survival of native insect species at risk (such as pollinators) by inadvertent viral spread; promoting species conservation; and increasing knowledge regarding insect viruses and their ecological and/or evolutionary relationships. 

## Figures and Tables

**Table 1:** Overview of the wasp viral transcript best hits identified by sequence similarity searches.

Wasp/Contigs	Virus	Segment	Query Size (nt)	Type	Query Coverage (%)	Identity (%)	E-Value	Closest Sequence in GenBank	Accession
***A. compressa***	*Ampulexvirus narnaviri*	single	3732	nt	22	67	3.00 × 10^−5^	*Xiangshan narna-like virus*	OK491482.1
Ac_Contig1				aa	68	51	0.0	*Xiangshan narna-like virus*	UDL13948.1
***C. vestalis***	*Cotesiavirus virgavi*	single	9075	nt	1	76	4.00 × 10^−9^	*Abisko virus*	KY662294.1
Cv_Contig1				aa	31	40	0.0	*Sanya virga-like virus 1*	UHM27517.1
Cv_RNA_ segment_1	*Cotesiavirus orthomyxi*	1	2499	aa	94	48	0.0	*Phasmatodean orthomyxo-related virus OKIAV172*	QMP82248.1
Cv_RNA_ segment_2		2	2470	aa	92	35	3.00 × 10^−132^	*Phasmatodean orthomyxo-related virus OKIAV172*	QMP82297.1
Cv_RNA_ segment_3		3	2256	aa	93	36	5.00 × 10^−148^	*Hymenopteran orthomyxo-related virus OKIAV171*	QPL15315.1
Cv_RNA_ segment_4		4	1577	aa	83	27	9.00 × 10^−45^	*Hymenopteran orthomyxo-related virus OKIAV173*	QMP82117.1
Cv_RNA_ segment_5		5	1518	aa	66	34	1.00 × 10^−21^	*Blattodean orthomyxo-related virus OKIAV181*	QMP82185.1
Cv_Contig2	*Cotesiavirus rhabdovi*	single	12,294	nt	4	70	5.00 × 10^−42^	*San Gabriel mononegavirus*	BK059423.1
				aa	51	43	0.0	*Hymenopteran rhabdo-related virus*	UOS86047.1
				aa	51	42	0.0	*Wuhan Ant Virus*	YP_009304559.1
Cv_Contig3	*Cotesiavirus chinense*	1	6636	nt	10	69	3.00 × 10^−16^	*Wuhan insect virus 16*	KX884733.1
				aa	92	41	0.0	*Wuhan insect virus 16*	APG79216.1
		2	1579	aa	67	29	1.00 × 10^−39^	*Hymenopteran phenui-related virus OKIAV282*	QMP82201.1
		3	868	aa	72	33	4.00 × 10^−26^	*Hymenopteran phenui-related virus OKIAV275*	QPL15371.1
***D. alloeum***	*Diachasmavirus* *michiganense*	single	11,634	nt	28	67	0.0	*Gudgenby Calliphora mononega-like virus*	MT129693.1
Da_Contig1				aa	51	51	0.0	*Gudgenby Calliphora mononega-like virus*	QIJ70030.1
Da_RNA_ segment_1	*Diachasmavirus* *orthomyxi*	1	2488	aa	85	32	5.00 × 10^−115^	*Phasmatodean orthomyxo-related virus OKIAV172*	QMP82297.1
Da_RNA_ segment_2		2	2456	aa	91	49	0.0	*Hymenopteran orthomyxo-related virus OKIAV173*	QMP82112.1
Da_RNA_ segment_3		3	2235	aa	95	37	1.00 × 10^−150^	*Hymenopteran orthomyxo-related virus OKIAV173*	QMP82372.1
Da_RNA_ segment_4		4	1803	aa	88	33	3.00 × 10^−82^	*Old quarry swamp virus*	AYP67576.1
Da_RNA_ segment_5		5	1586	aa	85	29	2.00 × 10^−40^	*Hymenopteran orthomyxo-related virus OKIAV173*	QMP82117.1
Da_Contig2	*Pterovirus diachasmae*	single	4108	aa	96	31	0.0	*Hymenopteran chu-related virus OKIAV147*	QPB73971.1
***E. lituratus***	*Alphahymrhavirus* *ectemnius*	single	12,682	nt	12	69	5.00 × 10^−111^	*Hymenopteran rhabdo-related virus OKIAV38*	MT153454.1
El_Contig1				aa	49	49	0.0	*Lasius neglectus virus 2*	AYW51543.1
***P. lugubris***									
Pl_Contig1	*Pemphredonvirus anglici*	single	5813	aa	50	43	0.0	*Megastigmus ssRNA virus*	QDZ71189.1
Pl_Contig2	*Betapartitivirus* *pemphredoni*	1	2397	nt	97	79	0.0	*Dill cryptic virus 2* *segment 1*	JX971984.1
Pl_Contig2.2		2	2271	nt	99	75	0.0	*Dill cryptic virus 2* *segment 2*	JX971985.1
Pl_Contig3	*Alphaendornavirus* *pemphredoni 1*	single	3462	nt	5	73	2.00 × 10^−21^	*Phaseolus lunatus alphaendornavirus*	MT792849.1
				aa	96	36	0.0	*Lily alphaendornavirus*	UPO25292.1
Pl_Contig4	*Alphaendornavirus* *pemphredoni 2*	single	11,115	aa	75	30	0.0	*Geranium carolinianum endornavirus*	QBB21108.1
Pl_Contig5	*Pemphredonvirus endornavi*	single	14,232	aa	80	33	0.0	*Hallsjon virus*	UYL94274.1
Pl_Contig6	*Mitovirus pemphredoni*	single	2141	aa	85	34	9.00 × 10^−107^	*Hangzhou altica cyanea mitovirus 1*	UHK03009.1
Pl_Contig7	*Unuamitovirus pemphredoni*	single	1056	aa	86	38	2.00 × 10^−82^	*Entomophthora muscae mitovirus 2*	QCF24453.1
Pl_Contig10	*Hubei narna-like virus 25* isolate *P. lugubris*	single	1143	nt	100	92	0.0	*Hubei narna-like virus 25* strain SCM51430	KX883546.1
***T. podisi*** Tp_Contig1	*Halyomorpha halys virus* isolate *T. podisi*	single	8285	nt	100	98	0.0	*Halyomorpha halys virus* isolate Beltsville	KF699344.1

## Data Availability

The nucleotide sequence data reported in this work are available from the Third Party Annotation Section of the DDBJ/ENA/GenBank databases under the accession numbers TPA: BK063681-BK063709.

## Supplementary Materials

The following supporting information can be downloaded at: https://www.mdpi.com/article/10.3390/v15122448/s1. Figure S1: Geographical distribution of the libraries used in the study classified by wasp species; Figure S2: Metagenomics analysis grouped by species of parasitoid wasps; Figure S3: Metagenomics analysis for each RNA-seq library included in the study; Figure S4: Characterization of segments, ORFs, and domains of sequences closely related to the assembled viral transcripts identified in the work; Table S1: Overview of RNA-deep sequenced libraries used in the work. Table S2: Detailed result of sequence similarity searches of transcripts related to viral species. Table S3: Description of the parameters applied in the phylogenetic analyses.
