# Supplementary material for: The Viromes of Six Ecosystem Service Provider Parasitoid Wasps
Source: Viruses. 2023 Dec 16;15(12):2448. doi: 10.3390/v15122448 (PMC10747428; doi:10.3390/v15122448)
Supplement: Supplementary file 1 [file viruses-15-02448-s001.zip › Supplementary Information.pdf]

Supplementary Information

The Viromes of Six Ecosystem Service Providers Parasitoid Wasps

Gabriela B. Caldas-Garcia[1], Vinícius Castro Santos[2], Paula Luize Camargos Fonseca[1,3], João Paulo Pereira de Almeida[2]; Marco Antônio Costa[4], Eric Roberto Guimarães Rocha Aguiar [1, *1]

[1] Virus Bioinformatics laboratory, Centro de Biotecnologia e Genética, Universidade Estadual de Santa Cruz, Ilhéus 45662-900, Brazil; gabrielabcg@gmail.com (G.B.C.-G.); camargos.paulaluize@gmail.com (P.L.C.F.)

[2] Department of Biochemistry and Immunology, Instituto de Ciências Biológicas, Universidade Federal de Minas Gerais, Belo Horizonte 30270-901, Brazil; vini8cs@gmail.com (V.C.S.); joaopaulobio@ufmg.br (J.P.P.d.A.)

[3] Department of Genetics, Instituto de Ciências Biológicas, Universidade Federal de Minas Gerais, Belo Horizonte 30270-901, Brazil

[4] Departament of Biological Sciences, Universidade Estadual de Santa Cruz, Ilhéus 45662-900, Brazil; costama@uesc.br

* Correspondence: ericgdp@gmail.com

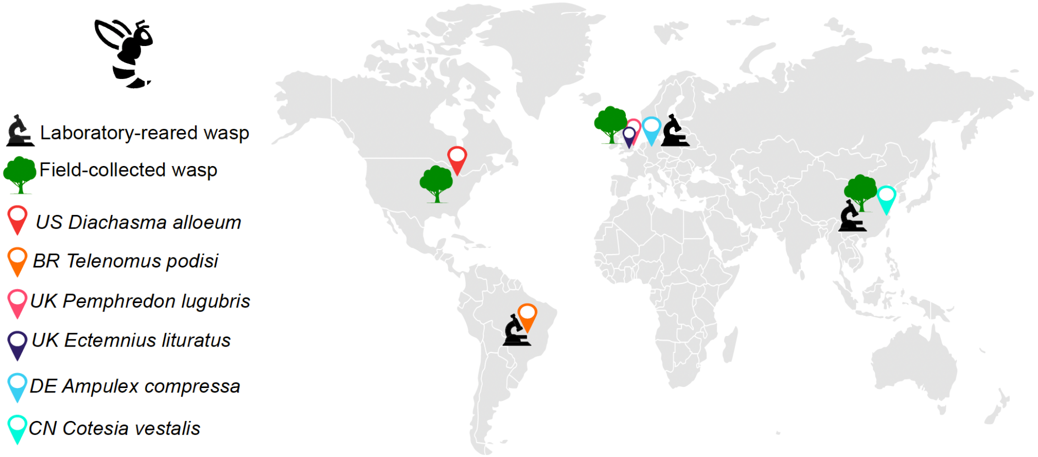

Supplementary Figure S1. Geographical distribution of libraries used in the study classified by wasp species.

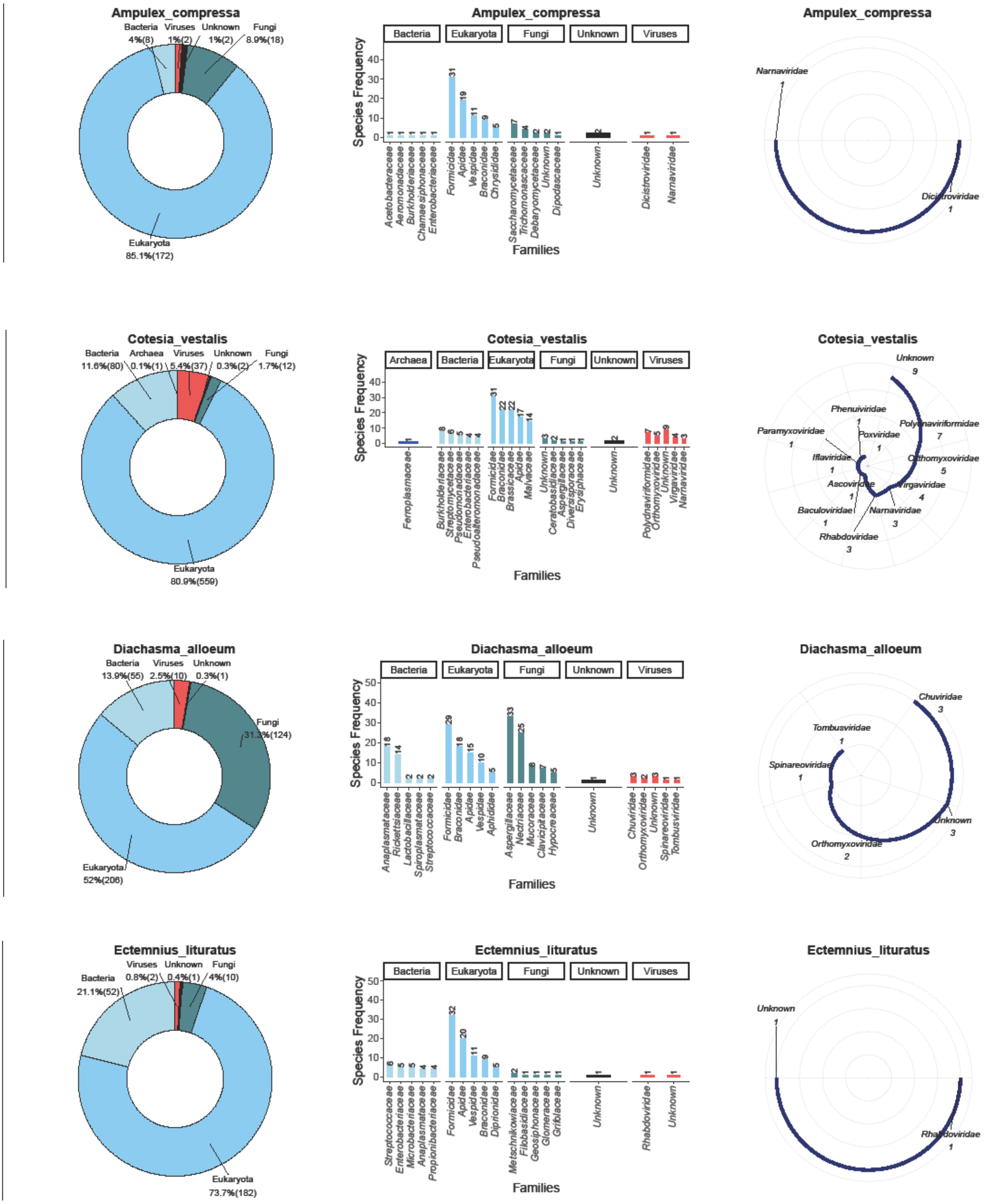

Ampulex_compressa
Bacteria 4%(8)
Viruses 1%(2)
Unknown 1%(2)
Fungi 8.9%(18)
Eukaryota 85.1%(172)
Bacteria
Eukaryota
Fungi
Unknown
Viruses
Species Frequency
Families
Acetobacteraceae
Aeromonadaceae
Burkholderiaceae
Chamaesiphonaceae
Enterobacteriaceae
Formicidae
Apidae
Vespidae
Braconidae
Chrysididae
Saccharomycetaceae
Trichomonascaceae
Debaryomycetaceae
Unknown
Dipodascaceae
Dicistroviridae
Narnaviridae
Narnaviridae 1
Dicistroviridae 1
Cotesia_vestalis
Bacteria 11.6%(80)
Archaea 0.1%(1)
Viruses 5.4%(37)
Unknown 0.3%(2)
Fungi 1.7%(12)
Eukaryota 80.9%(559)
Archaea
Ferroplasmaceae
Burkholderiaceae
Streptomycetaceae
Pseudomonadaceae
Enterobacteriaceae
Pseudoalteromonadaceae
Formicidae
Braconidae
Brassicaceae
Apidae
Malvaceae
Ceratobasidiaceae
Aspergillaceae
Diversisporaceae
Erysiphaceae
Polydnaviriformidae
Orthomyxoviridae
Virgaviridae
Narnaviridae
Unknown 9
Phenuiviridae 1
Polydnaviriformidae 7
Paramyxoviridae 1
Poxviridae 1
Iflaviridae 1
Orthomyxoviridae 5
Ascoviridae 1
Virgaviridae 4
Baculoviridae 1
Narnaviridae 3
Rhabdoviridae 3
Diachasma_alloeum
Bacteria 13.9%(55)
Viruses 2.5%(10)
Unknown 0.3%(1)
Fungi 31.3%(124)
Eukaryota 52%(206)
Anaplasmataceae
Rickettsiaceae
Lactobacillaceae
Spiroplasmataceae
Streptococcaceae
Formicidae
Braconidae
Apidae
Vespidae
Aphididae
Aspergillaceae
Nectriaceae
Mucoraceae
Clavicipitaceae
Hypocreaceae
Chuviridae
Orthomyxoviridae
Spinareoviridae
Tombusviridae
Chuviridae 3
Tombusviridae 1
Spinareoviridae 1
Unknown 3
Orthomyxoviridae 2
Ectemnius_lituratus
Bacteria 21.1%(52)
Viruses 0.8%(2)
Unknown 0.4%(1)
Fungi 4%(10)
Eukaryota 73.7%(182)
Streptococcaceae
Enterobacteriaceae
Microbacteriaceae
Anaplasmataceae
Propionibacteriaceae
Formicidae
Apidae
Vespidae
Braconidae
Diprionidae
Metschnikowiaceae
Fibbasidiaceae
Geosiphonaceae
Glomeraceae
Gribiaceae
Rhabdoviridae
Unknown 1
Rhabdoviridae 1

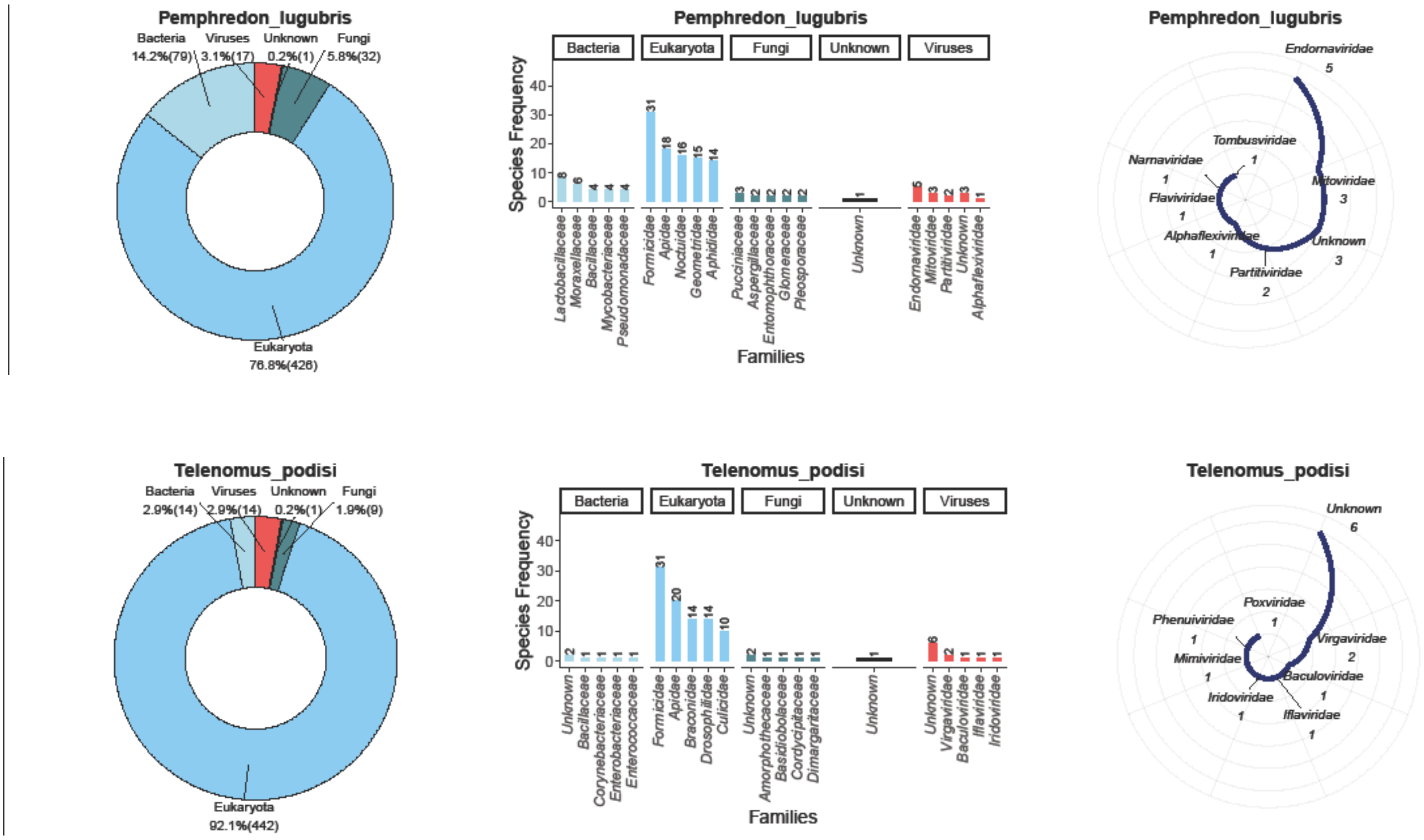

Supplementary Figure S2. Metagenomics analysis grouped by species of parasitoid wasps. In order: *A. compressa, C. vestalis, D. alloeum, E. lituratus, P. lugubris, and T. podisi*. On the left, donut plot showing percentages of organisms' diversity found by kingdom. The categories are Eukaryota, Bacteria, Fungi, Viruses, and Unknown. On the middle, bar charts with details on the families within each kingdom (x-axis) and the species frequency by family (y-axis). On the right, radar plot showing the diversity of viral families by parasitoid species.

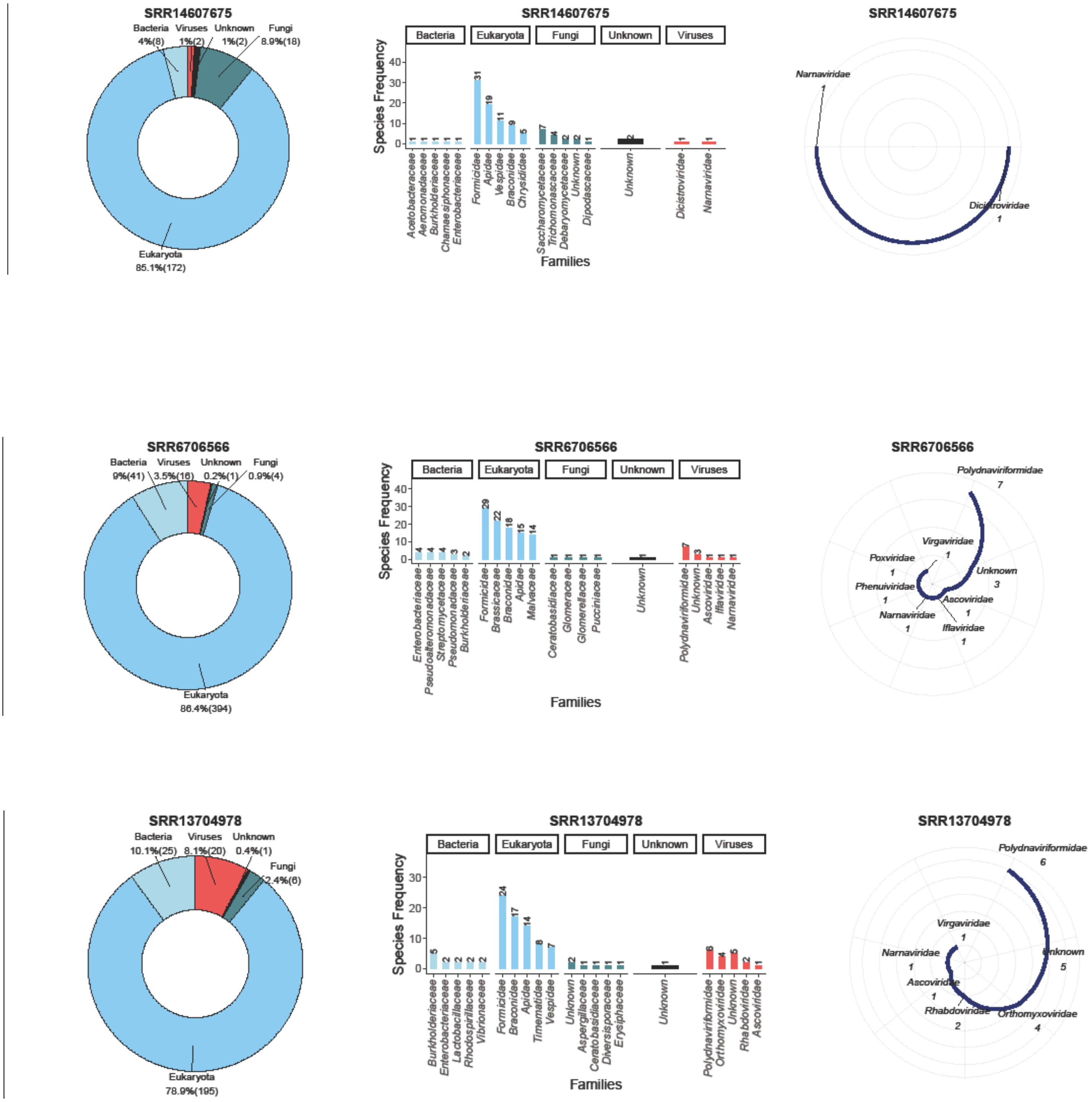

SRR14607675
Bacteria 4%(8)
Viruses 1%(2)
Unknown 1%(2)
Fungi 8.9%(18)
Eukaryota 85.1%(172)
SRR14607675
Bacteria
Eukaryota
Fungi
Unknown
Viruses
Species Frequency
Acetobacteraceae
Aeromonadaceae
Burkholderiaceae
Chamaesiphonaceae
Enterobacteriaceae
Formicidae
Apidae
Vespidae
Braconidae
Chrysididae
Saccharomycetaceae
Trichomonascaceae
Debaryomycetaceae
Unknown
Dipodascaceae
Unknown
Dicistroviridae
Narnaviridae
Families
SRR14607675
Narnaviridae 1
Dicistroviridae 1
SRR6706566
Bacteria 9%(41)
Viruses 3.5%(16)
Unknown 0.2%(1)
Fungi 0.9%(4)
Eukaryota 86.4%(394)
SRR6706566
Bacteria
Eukaryota
Fungi
Unknown
Viruses
Species Frequency
Enterobacteriaceae
Pseudoalteromonadaceae
Streptomycetaceae
Pseudomonadaceae
Burkholderiaceae
Formicidae
Brassicaceae
Braconidae
Apidae
Malvaceae
Ceratobasidiaceae
Glomeraceae
Glomerellaceae
Pucciniaceae
Unknown
Polydnaviriformidae
Unknown
Ascoviridae
Iflaviridae
Narnaviridae
Families
SRR6706566
Polydnaviriformidae 7
Virgaviridae 1
Poxviridae 1
Unknown 3
Phenuiviridae 1
Ascoviridae 1
Narnaviridae 1
Iflaviridae 1
SRR13704978
Bacteria 10.1%(25)
Viruses 8.1%(20)
Unknown 0.4%(1)
Fungi 2.4%(6)
Eukaryota 78.9%(195)
SRR13704978
Bacteria
Eukaryota
Fungi
Unknown
Viruses
Species Frequency
Burkholderiaceae
Enterobacteriaceae
Lactobacillaceae
Rhodospirillaceae
Vibrionaceae
Formicidae
Braconidae
Apidae
Timematidae
Vespidae
Unknown
Aspergillaceae
Ceratobasidiaceae
Diversisporaceae
Erysiphaceae
Unknown
Polydnaviriformidae
Orthomyxoviridae
Unknown
Rhabdoviridae
Ascoviridae
Families
SRR13704978
Polydnaviriformidae 6
Virgaviridae 1
Narnaviridae 1
Unknown 5
Ascoviridae 1
Rhabdoviridae 2
Orthomyxoviridae 4

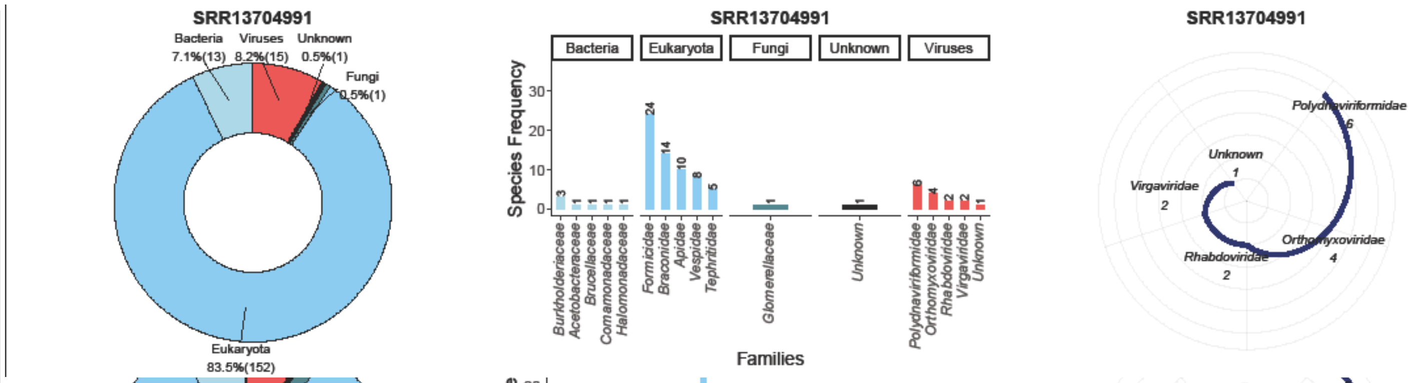
SRR13704991
Bacteria 7.1%(13)
Viruses 8.2%(15)
Unknown 0.5%(1)
Fungi 0.5%(1)
Eukaryota 83.5%(152)
SRR13704991
Bacteria
Eukaryota
Fungi
Unknown
Viruses
Species Frequency
Burkholderiaceae
Acetobacteraceae
Brucellaceae
Comamonadaceae
Halomonadaceae
Formicidae
Braconidae
Apidae
Vespidae
Tephritidae
Glomerellaceae
Unknown
Polydnaviriformidae
Orthomyxoviridae
Rhabdoviridae
Virgaviridae
Unknown
Families
SRR13704991
Polydnaviriformidae 6
Unknown 1
Virgaviridae 2
Orthomyxoviridae 4
Rhabdoviridae 2

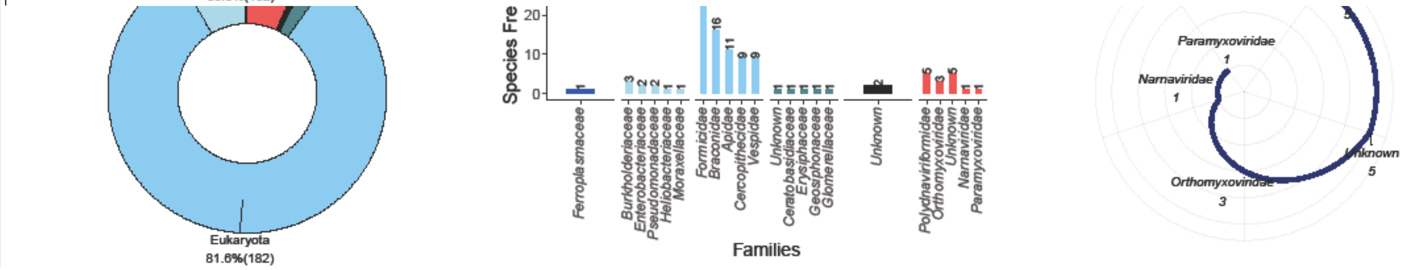
Eukaryota 81.6%(182)
Species Fre
Ferroplasmaceae
Burkholderiaceae
Enterobacteriaceae
Pseudomonadaceae
Helicobacteraceae
Moraxellaceae
Formicidae
Braconidae
Apidae
Cercopithecidae
Vespidae
Unknown
Ceratobasidiaceae
Erysiphaceae
Geosiphonaceae
Glomerellaceae
Unknown
Polydnaviriformidae
Orthomyxoviridae
Unknown
Narnaviridae
Paramyxoviridae
Families
Paramyxoviridae 1
Narnaviridae 1
Unknown 5
Orthomyxoviridae 3

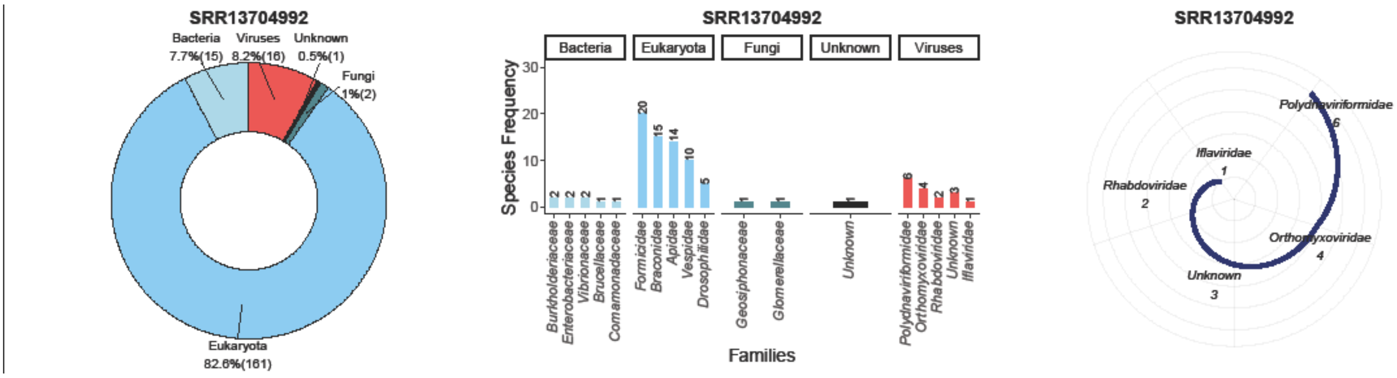
SRR13704992
Bacteria 7.7%(15)
Viruses 8.2%(16)
Unknown 0.5%(1)
Fungi 1%(2)
Eukaryota 82.6%(161)
SRR13704992
Bacteria
Eukaryota
Fungi
Unknown
Viruses
Species Frequency
Burkholderiaceae
Enterobacteriaceae
Vibrionaceae
Brucellaceae
Comamonadaceae
Formicidae
Braconidae
Apidae
Vespidae
Drosophilidae
Geosiphonaceae
Glomerellaceae
Unknown
Polydnaviriformidae
Orthomyxoviridae
Rhabdoviridae
Unknown
Iflaviridae
Families
SRR13704992
Polydnaviriformidae 6
Iflaviridae 1
Rhabdoviridae 2
Orthomyxoviridae 4
Unknown 3

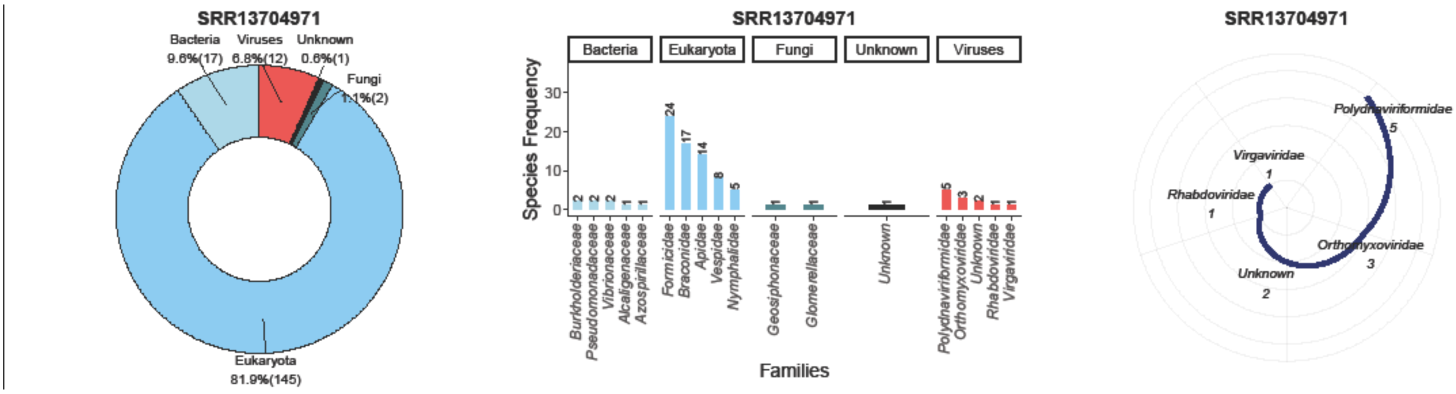
SRR13704971
Bacteria 9.6%(17)
Viruses 6.8%(12)
Unknown 0.6%(1)
Fungi 1.1%(2)
Eukaryota 81.9%(145)
SRR13704971
Bacteria
Eukaryota
Fungi
Unknown
Viruses
Species Frequency
Burkholderiaceae
Pseudomonadaceae
Vibrionaceae
Alcaligenaceae
Azospirillaceae
Formicidae
Braconidae
Apidae
Vespidae
Nymphalidae
Geosiphonaceae
Glomerellaceae
Unknown
Polydnaviriformidae
Orthomyxoviridae
Unknown
Rhabdoviridae
Virgaviridae
Families
SRR13704971
Polydnaviriformidae 5
Virgaviridae 1
Rhabdoviridae 1
Orthomyxoviridae 3
Unknown 2

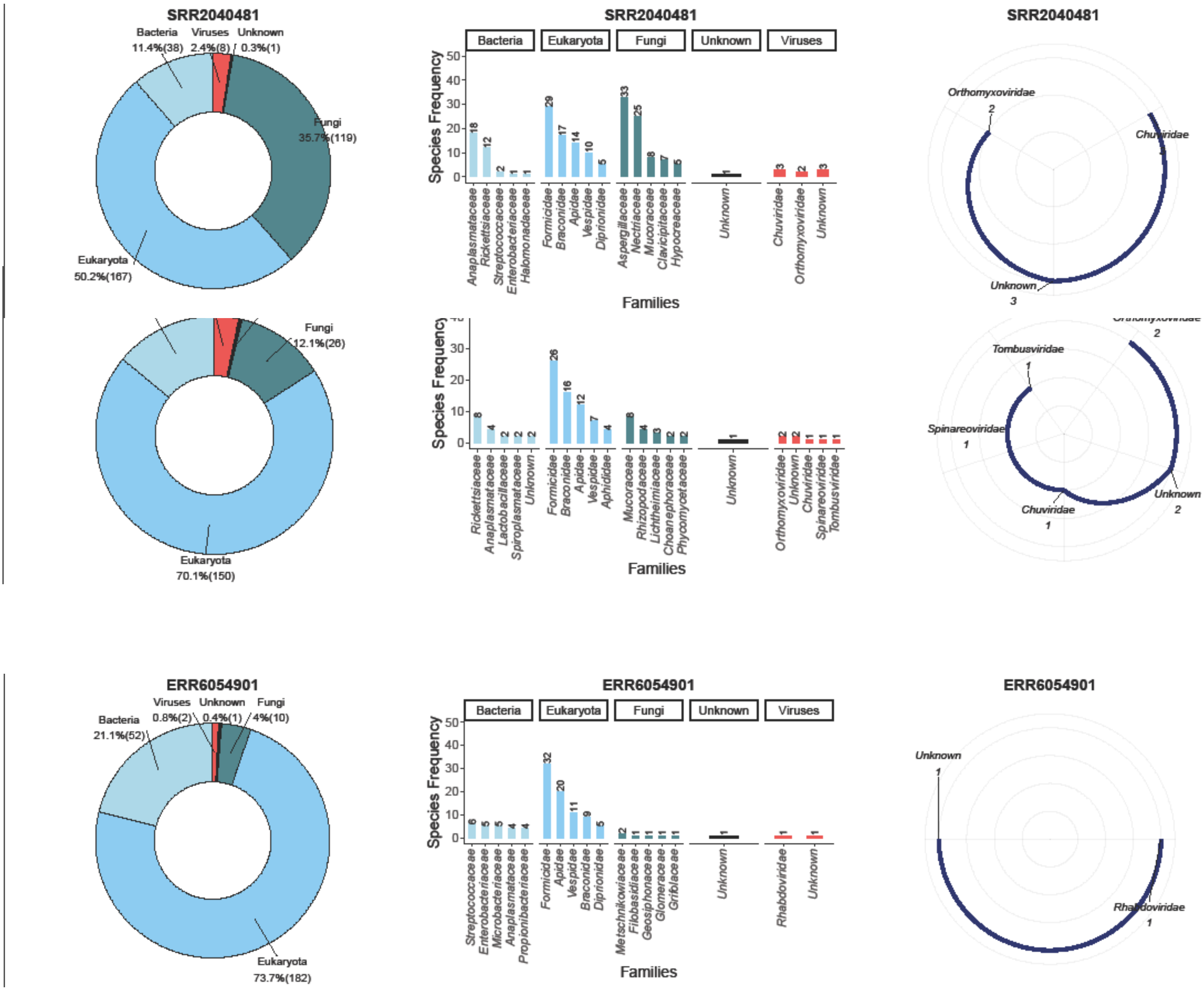
SRR2040481
Bacteria
11.4%(38)
Viruses
2.4%(8)
Unknown
0.3%(1)
Fungi
35.7%(119)
Eukaryota
50.2%(167)
SRR2040481
Bacteria
Eukaryota
Fungi
Unknown
Viruses
Species Frequency
Anaplasmataceae
Rickettsiaceae
Streptococcaceae
Enterobacteriaceae
Halomonadaceae
Formicidae
Braconidae
Apidae
Vespidae
Diprionidae
Aspergillaceae
Nectriaceae
Mucoraceae
Clavicipitaceae
Hypocreaceae
Unknown
Chuviridae
Orthomyxoviridae
Unknown
Families
SRR2040481
Orthomyxoviridae
2
Chuviridae
Unknown
3
Fungi
12.1%(26)
Eukaryota
70.1%(150)
Species Frequency
Rickettsiaceae
Anaplasmataceae
Lactobacillaceae
Spiroplasmataceae
Unknown
Formicidae
Braconidae
Apidae
Vespidae
Aphididae
Mucoraceae
Rhizopodaceae
Lichtheimiaceae
Choanephoraceae
Phycomycetaceae
Unknown
Orthomyxoviridae
Unknown
Chuviridae
Spinareoviridae
Tombusviridae
Families
Tombusviridae
1
Spinareoviridae
1
Chuviridae
1
Unknown
2
ERR6054901
Bacteria
21.1%(52)
Viruses
0.8%(2)
Unknown
0.4%(1)
Fungi
4%(10)
Eukaryota
73.7%(182)
ERR6054901
Bacteria
Eukaryota
Fungi
Unknown
Viruses
Species Frequency
Streptococcaceae
Enterobacteriaceae
Microbacteriaceae
Anaplasmataceae
Propionibacteriaceae
Formicidae
Apidae
Vespidae
Braconidae
Diprionidae
Metschnikowiaceae
Filobasidiaceae
Geosiphonaceae
Glomeraceae
Gribiaceae
Unknown
Rhabdoviridae
Unknown
Families
ERR6054901
Unknown
1
Rhabdoviridae
1

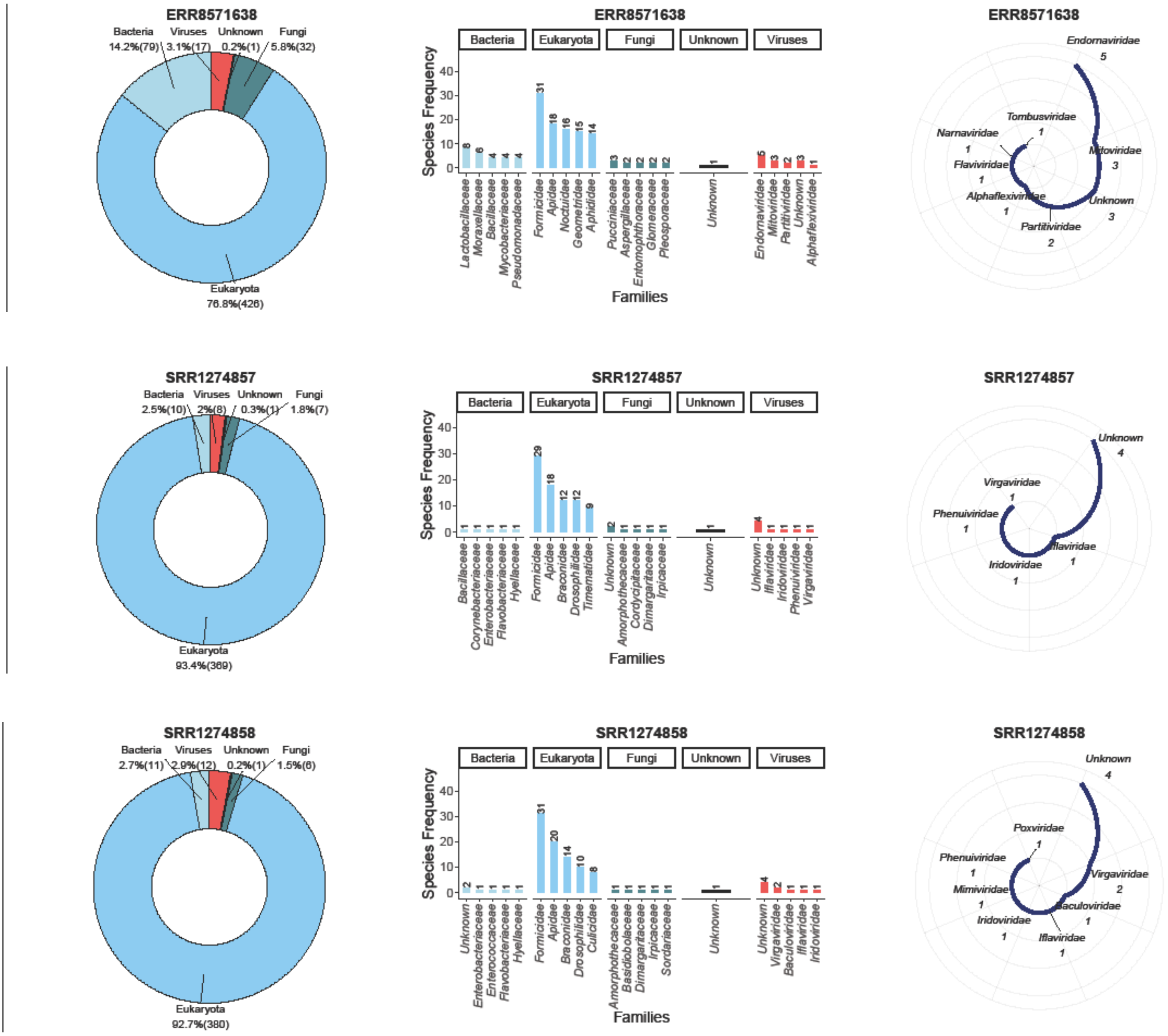

Supplementary Figure S3. Metagenomics analysis for each RNA-seq library included in the study. In order: SRR14607675 (*A. compressa*); SRR6706566, SRR13704978, SRR13704979, SRR13704980, SRR13704991, SRR13704992, SRR13704971 *(C. vestalis);* SRR2041626, SRR2040481 *(D. alloeum);* ERR6054901 *(E. lituratus);* ERR8571638 *(P. lugubris); and* SRR1274857, SRR1274858 *(T. podisi).* On the left, donut plot showing percentages of organisms' diversity found by kingdom. The categories are Eukaryota, Bacteria, Fungi, Viruses, and Unknown. On the middle, bar charts with details on the families within each kingdom (x-axis) and the species frequency by family (y-axis). On the right, radar plot showing the diversity of viral families by parasitoid species.

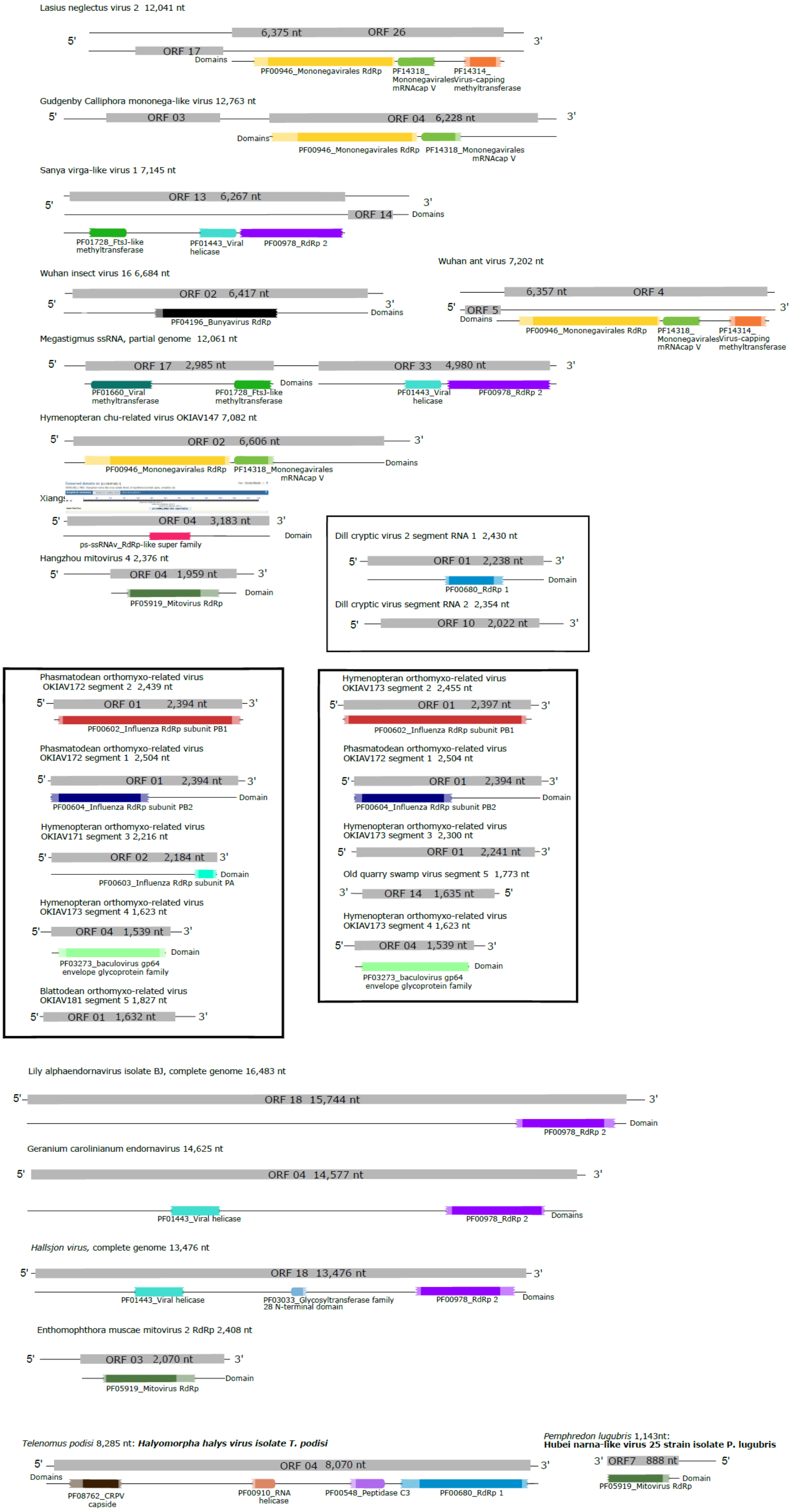

Lasius neglectus virus 2 12,041 nt
5'
6,375 nt ORF 26
3'
ORF 17
Domains
PF00946_Mononegavirales RdRp
PF14318_ Mononegavirales mRNAcap V
PF14314_ Virus-capping methyltransferase
Gudgenby Calliphora mononega-like virus 12,763 nt
5'
ORF 03
ORF 04 6,228 nt
3'
Domains
PF00946_Mononegavirales RdRp
PF14318_Mononegavirales mRNAcap V
Sanya virga-like virus 1 7,145 nt
ORF 13 6,267 nt
5'
3'
ORF 14
Domains
PF01728_FtsJ-like methyltransferase
PF01443_Viral helicase
PF00978_RdRp 2
Wuhan ant virus 7,202 nt
Wuhan insect virus 16 6,684 nt
ORF 02 6,417 nt
5'
3'
Domains
PF04196_Bunyavirus RdRp
6,357 nt ORF 4
5'
ORF 5
3'
Domains
PF00946_Mononegavirales RdRp
PF14318_ Mononegavirales mRNAcap V
PF14314_ Virus-capping methyltransferase
Megastigmus ssRNA, partial genome 12,061 nt
5'
ORF 17 2,985 nt
ORF 33 4,980 nt
3'
Domains
PF01660_Viral methyltransferase
PF01728_FtsJ-like methyltransferase
PF01443_Viral helicase
PF00978_RdRp 2
Hymenopteran chu-related virus OKIAV147 7,082 nt
5'
ORF 02 6,606 nt
3'
Domains
PF00946_Mononegavirales RdRp
PF14318_Mononegavirales mRNAcap V
Xiang
5'
ORF 04 3,183 nt
3'
Domain
ps-ssRNAv_RdRp-like super family
Hangzhou mitovirus 4 2,376 nt
5'
ORF 04 1,959 nt
3'
Domain
PF05919_Mitovirus RdRp
Dill cryptic virus 2 segment RNA 1 2,430 nt
5'
ORF 01 2,238 nt
3'
Domain
PF00680_RdRp 1
Dill cryptic virus segment RNA 2 2,354 nt
5'
ORF 10 2,022 nt
3'
Phasmatodean orthomyxo-related virus OKIAV172 segment 2 2,439 nt
5'
ORF 01 2,394 nt
3'
PF00602_Influenza RdRp subunit PB1
Phasmatodean orthomyxo-related virus OKIAV172 segment 1 2,504 nt
5'
ORF 01 2,394 nt
3'
Domain
PF00604_Influenza RdRp subunit PB2
Hymenopteran orthomyxo-related virus OKIAV171 segment 3 2,216 nt
5'
ORF 02 2,184 nt
3'
Domain
PF00603_Influenza RdRp subunit PA
Hymenopteran orthomyxo-related virus OKIAV173 segment 4 1,623 nt
5'
ORF 04 1,539 nt
3'
Domain
PF03273_baculovirus gp64 envelope glycoprotein family
Blattodean orthomyxo-related virus OKIAV181 segment 5 1,827 nt
5'
ORF 01 1,632 nt
3'
Hymenopteran orthomyxo-related virus OKIAV173 segment 2 2,455 nt
5'
ORF 01 2,397 nt
3'
PF00602_Influenza RdRp subunit PB1
Phasmatodean orthomyxo-related virus OKIAV172 segment 1 2,504 nt
5'
ORF 01 2,394 nt
3'
Domain
PF00604_Influenza RdRp subunit PB2
Hymenopteran orthomyxo-related virus OKIAV173 segment 3 2,300 nt
5'
ORF 01 2,241 nt
3'
Old quarry swamp virus segment 5 1,773 nt
3'
ORF 14 1,635 nt
5'
Hymenopteran orthomyxo-related virus OKIAV173 segment 4 1,623 nt
5'
ORF 04 1,539 nt
3'
Domain
PF03273_baculovirus gp64 envelope glycoprotein family
Lily alphaendornavirus isolate BJ, complete genome 16,483 nt
5'
ORF 18 15,744 nt
3'
Domain
PF00978_RdRp 2
Geranium carolinianum endornavirus 14,625 nt
5'
ORF 04 14,577 nt
3'
PF01443_Viral helicase
PF00978_RdRp 2
Domains
Hallsjon virus, complete genome 13,476 nt
5'
ORF 18 13,476 nt
3'
PF01443_Viral helicase
PF03033_Glycosyltransferase family 28 N-terminal domain
PF00978_RdRp 2
Domains
Enthomophthora muscae mitovirus 2 RdRp 2,408 nt
5'
ORF 03 2,070 nt
3'
Domain
PF05919_Mitovirus RdRp
Telenomus podisi 8,285 nt: Halyomorpha halys virus isolate T. podisi
5'
ORF 04 8,070 nt
3'
Domains
PF08762_CRPV capside
PF00910_RNA helicase
PF00548_Peptidase C3
PF00680_RdRp 1
Pemphredon lugubris 1,143nt:
Hubei narna-like virus 25 strain isolate P. lugubris
3'
ORF7 888 nt
5'
Domain
PF05919_Mitovirus RdRp

Supplementary Figure S4. Characterization of segments, ORFs and domains of the closely related sequences to the assembled viral transcripts identified in the work. All viruses represented in this figure are previously described viruses.
